# Supplementary material for: Shared decision-making for non-operative management versus operative management of hip fractures in selected frail older adults with a limited life expectancy: a protocol for a nationwide implementation study
Source: BMJ Open. 2024 Apr 17;14(4):e083429. doi: 10.1136/bmjopen-2023-083429 (PMC11029367; doi:10.1136/bmjopen-2023-083429)
Supplement: Supplementary data [file bmjopen-2023-083429supp001.pdf]

Online supplementary materials 1

Table S1: Contributors in NOM-Implementation study group

Institution	Location	First name	Last name	Title/Academic degree	Medical specialty
Alrijne ziekenhuis	Leiderdorp	E.	Sohl	MD PhD	Geriatrics
Alrijne ziekenhuis	Leiderdorp	J.A.	Jansen	MD	Orthopedics
Alrijne ziekenhuis	Leiderdorp	M.	Leijnen	MD PhD	Surgery
Carnisius Wilhelmina ziekenhuis	Nijmegen	W.A.H.	Van der Stappen	MD	Surgery
Carnisius Wilhelmina ziekenhuis	Nijmegen	J.	Jansen	MD PhD	Orthopedics
Carnisius Wilhelmina ziekenhuis	Nijmegen	M.	Peters-Kop	PA	Geriatric trauma unit
Catharina ziekenhuis	Eindhoven	A.H.	Van der Veen	MD PhD	Surgery
Catharina ziekenhuis	Eindhoven	N.C.	Schepel	MD	Orthopedics
Catharina ziekenhuis	Eindhoven	J.A.M.	Wilmer	MD	Geriatrics
Dijklander ziekenhuis	Hoorn	J.	Steens	MD PhD	Orthopedics
Dijklander ziekenhuis	Hoorn	J.	Winkelhagen	MD	Surgery
Dijklander ziekenhuis	Hoorn	N.M.F.	Noorda	MD	Geriatrics
Elisabeth TweeSteden ziekenhuis	Tilburg	H.A.A.M.	Maas	MD PhD	Geriatrics
Franciscus ziekenhuis	Rotterdam	O.	Wijers	MD PhD	Surgery
Franciscus ziekenhuis	Rotterdam	V.	Vis	PA	Geriatric trauma unit
Maasstad ziekenhuis	Rotterdam	D.	Van der Stap	MD	Geriatrics
Maasstad ziekenhuis	Rotterdam	G.R.	Roukema	MD PhD	Surgery
Martini ziekenhuis	Groningen	E.	Bosma	MD	Surgery
Martini ziekenhuis	Groningen	T.M.	Van Raaij	MD PhD	Orthopedics
Martini ziekenhuis	Groningen	A.M.	Van der Knaap	MD	Geriatrics
Medisch spectrum Twente	Enschede	R.	de Groot	MD PhD	Surgery
Medisch spectrum Twente	Enschede	A.V.C.M.	Zeegers	MD PhD	Orthopedics
OLVG	Amsterdam	H.A.	Formijne Jonkers	MD PhD	Surgery
OLVG	Amsterdam	D.H.R.	Kempen	MD PhD	Orthopedics
OLVG	Amsterdam	K.	De Vries	MD	Geriatrics
Rijnstate ziekenhuis	Arnhem	A.F.	Pull ter Gunne	MD PhD	Surgery
Rijnstate ziekenhuis	Arnhem	M.P.	Somford	MD PhD	Orthopedics
Rode Kruis ziekenhuis	Beverwijk	E.	Tanis	MD PhD	Surgery
Rode Kruis ziekenhuis	Beverwijk	J.H.	Duits	MD	Geriatrics
Rode Kruis ziekenhuis	Beverwijk	R.A.	Zandbergen	MD	Orthopedics
St. Antonius ziekenhuis	Nieuwegein	N.C.	Leegwater	MD PhD	Orthopedics

St. Antonius ziekenhuis	Nieuwegein	O.C.	Geraghty	MD PhD	Geriatrics
Ziekenhuisgroep Twente	Almelo	J.H.	Hegeman	MD PhD	Surgery
Ziekenhuisgroep Twente	Almelo	E.M.	Regtuijt	MD	Geriatrics

MD, Doctor of medicine; PA, Physician assistant; PhD, Doctor of philosophy
