# Supplementary material for: Shared decision-making for non-operative management versus operative management of hip fractures in selected frail older adults with a limited life expectancy: a protocol for a nationwide implementation study
Source: BMJ Open. 2024 Apr 17;14(4):e083429. doi: 10.1136/bmjopen-2023-083429 (PMC11029367; doi:10.1136/bmjopen-2023-083429)
Supplement: Supplementary data [file bmjopen-2023-083429supp002.pdf]

Datum (dd-mm-jjjj): ___ - ___ - 20___

Datasheet interviews phase 1

Introductie:

- Deelnemer(s) welkom heten en bedanken voor hun deelname.
- Doel van het interview toelichten.
- Benoemen dat deelname betekent dat de antwoorden gebruikt zullen worden om het PNOM-handleiding waar mogelijk te verbeteren. Dit zal te allen tijden anoniem zijn. Wie dit niet wil, de kans geven om het gesprek te beëindigen of de bijeenkomst te verlaten.

Om PNOM te implementeren zijn naast de FRAIL-HIP studie 2 zijstudies uitgevoerd.

1) Enquête van Spronk *et al.* in 2022 onder 271 zorgprofessionals in het ziekenhuis. Dit heeft geleid tot 5 belemmerende factoren en 23 bevorderende factoren voor PNOM implementatie.
2) Interviews van Nijdam *et al.* in 2022 onder nabestaanden van patiënten bij wie voor PNOM gekozen was.

Hebt u aanvullingen of suggesties na lezen van de conceptversie? Zo ja, zou u die hieronder willen vermelden?

A) Vertegenwoordigers huisartsen:

B) Vertegenwoordigers SOGs:

C) Vertegenwoordigers KBO-PCOB:

Zeelenberg ML, *et al. BMJ Open* 2024; 14:e083429. doi: 10.1136/bmjopen-2023-083429

Datum (dd-mm-jjjj): ___ - ___ - 20___

Datasheet interviews phase 2

Introductie:
- Deelnemer(s) welkom heten en bedanken voor hun deelname.
- Doel van het interview toelichten.
- Benoemen dat deelname betekent dat de antwoorden gebruikt zullen worden om de PNOM-handleiding waar mogelijk te verbeteren. Dit zal te allen tijden anoniem zijn. Wie dit niet wil, de kans geven om het gesprek te beëindigen of de bijeenkomst te verlaten.

Om PNOM te implementeren is een PNOM-handleiding gemaakt. Dit is gebaseerd op de resultaten van de FRAIL-HIP en nevenstudies naar beperkende en bevorderende factoren voor implementatie. Het concept protocol bevat o.a.:
1) Doel van de implementatie
2) Benodigd lokaal team
3) Procedure om implementatie te bewerkstelligen
4) Factsheet
5) Data die nodig zijn om het effect van de implementatie te evalueren

Hebt u aanvullingen of suggesties na lezen van de conceptversie? Zo ja, zou u die hieronder willen vermelden?

A) Input voor PNOM-handleiding:

B) Input voor patiëntinformatie:

C) Input voor zakkaart:

Zeelenberg ML, *et al. BMJ Open* 2024; 14:e083429. doi: 10.1136/bmjopen-2023-083429

Datum (dd-mm-jjjj): ___ - ___ - 20___

D) Input voor factsheet:

____
____
____
____
____

E) Overig input:

____
____
____
____
____

Zeelenberg ML, *et al. BMJ Open* 2024; 14:e083429. doi: 10.1136/bmjopen-2023-083429

Datum (dd-mm-jjjj): ___ - ___ - 20___

Datasheet interviews phase 3 (nul-meting)

Introductie:
- Deelnemer(s) welkom heten en bedanken voor hun deelname.
- Doel van het interview toelichten.
- Benoemen dat deelname betekent dat de antwoorden gebruikt zullen worden om het PNOM protocol waar mogelijk te verbeteren. Dit zal te allen tijden anoniem zijn. Wie dit niet wil, de kans geven om het gesprek te beëindigen of de bijeenkomst te verlaten.

Uw ziekenhuis gaat deelnemen/neemt deel aan implementatie van PNOM voor een specifieke groep kwetsbare ouderen met een heupfractuur. Het protocol bevat o.a.:
1) Doel van de implementatie
2) Benodigd lokaal team
3) Procedure om implementatie te bewerkstelligen
4) Factsheet
5) Data die nodig zijn om het effect van de implementatie te evalueren

Eerst willen we graag inzicht in de huidige werkwijze.

1. Bent u gewend om SDM toe te passen, waarbij (uitgebreid) aandacht wordt besteed aan PNOM?
   - ☐ Ja, altijd
   - ☐ Ja, soms
   - ☐ Nee

2. Indien nee: waarom niet: ______________________
   ____

3. Indien ja: hoe vaak in het afgelopen jaar is PNOM middels SDM besproken?
   - ☐ 1 of 2 keer
   - ☐ 3 tot 5 keer
   - ☐ 5 tot 10 keer
   - ☐ 10 keer of vaker

4. Hoe vaak is daarbij voor PNOM gekozen?
   - ☐ 1 of 2 keer
   - ☐ 3 tot 5 keer
   - ☐ 5 tot 10 keer
   - ☐ 10 keer of vaker

5. Indien ja, wat wordt er bij SDM besproken over NOM? ______________________
   ____

6. Indien ja, wat was uw ervaring met PNOM? ______________________
   ____

Versie 1.0, d.d. 11 april 2023 Pagina 1 van 3

BMJ Publishing Group Limited (BMJ) disclaims all liability and responsibility arising from any reliance placed on this supplemental material which has been supplied by the author(s)

Datum (dd-mm-jjjj): ___ - ___ - 20___

Wat vindt u van de huidige versie van de PNOM-handleiding? Hebt u aanvullingen of suggesties na lezen ervan? Zo ja, zou u die hieronder willen vermelden?

A) Input voor PNOM-handleiding:

B) Input voor patiëntinformatie:

C) Input voor zakkaart:

D) Input voor factsheet:

E) Overig input:

Zeelenberg ML, *et al. BMJ Open* 2024; 14:e083429. doi: 10.1136/bmjopen-2023-083429

Datum (dd-mm-jjjj): ___ - ___ - 20___

Tot slot willen we graag iets meer weten over uzelf.

1.	Leeftijd	☐ <30 jaar<br>☐ 30 t/m 39 jaar<br>☐ 40 t/m 49 jaar<br>☐ 50 t/m 59 jaar<br>☐ 60 jaar of ouder<br>☐ Wil niet zeggen
2.	Geslacht	☐ Man<br>☐ Vrouw<br>☐ Wil niet zeggen
3.	Functie	☐ Traumachirurg<br>☐ Orthopedisch chirurg<br>☐ Geriater<br>☐ Verpleegkundig specialist<br>☐ Physician assistant<br>☐ Manager, namelijk: ____________________<br>☐ Anders, namelijk: ____________________
4.	Religie / geloofsovertuiging	_________________________
5.	Heeft uw geloofsovertuiging effect op toepassen van PNOM	☐ Ja<br>☐ Nee<br>☐ Wil niet zeggen
6.	Actieve betrokkenheid bij PNOM na heupfractuur	☐ Ja<br>☐ Nee

Zeelenberg ML, *et al. BMJ Open* 2024; 14:e083429. doi: 10.1136/bmjopen-2023-083429

Datum (dd-mm-jjjj): ___ - ___ - 20___

Datasheet interviews phase 3 (follow-up)

Introductie:
- Deelnemer(s) welkom heten en bedanken voor hun deelname.
- Doel van het interview toelichten.
- Benoemen dat deelname betekent dat de antwoorden gebruikt zullen worden om het PNOM protocol waar mogelijk te verbeteren. Dit zal te allen tijden anoniem zijn. Wie dit niet wil, de kans geven om het gesprek te beëindigen of de bijeenkomst te verlaten.

Uw ziekenhuis neemt deel aan implementatie van PNOM voor een specifieke groep kwetsbare ouderen met een heupfractuur.

Eerst willen we graag weten wat u van de PNOM-implementatie vindt.

1. Hebt u het idee dat door de PNOM implementatie de zorg voor de doelgroep is verbeterd?
   ☐ Ja, de zorg is verbeterd
   ☐ Neutraal
   ☐ Nee, de zorg is verslechterd

2. Indien ja, wat is er verbeterd? ______________________________
   ____

3. Indien nee, wat is er verslechterd? ______________________________
   ____

Zeelenberg ML, *et al. BMJ Open* 2024; 14:e083429. doi: 10.1136/bmjopen-2023-083429

Datum (dd-mm-jjjj): ___ - ___ - 20___

Wat vindt u van de huidige versie van de PNOM-handleiding en was is uw ervaring tot nu toe? Als u aanvullingen of suggesties hebt, zou u die hieronder willen vermelden?

A) Dingen die goed gingen:

B) Dingen die minder/niet goed gingen en/of beter moeten:

C) Input voor PNOM-handleiding:

D) Input voor patiëntinformatie:

Versie 1.0, d.d. 11 april 2023

Datum (dd-mm-jjjj): ___ - ___ - 20___

E) Input voor zakkaart:

F) Input voor factsheet:

G) Overig input:

H) Benodigdheden om implementatie nog verder te verbeteren:

Zeelenberg ML, *et al. BMJ Open* 2024; 14:e083429. doi: 10.1136/bmjopen-2023-083429

ID: PNOM-Implementatie - ____ - ____ **Datum (dd-mm-jjjj): ___ - ___ - 20___**

Introductie:

- Deelnemer(s) welkom heten en bedanken voor hun deelname.
- Doel van het interview toelichten.
- Benoemen dat deelname betekent dat de antwoorden gebruikt zullen worden in het onderzoek. Dit zal te allen tijden anoniem zijn. Als de gesprekspartner dat niet wil, wordt het gesprek beëindigd.

Tevredenheid van patiënt met de gekozen behandeling

Nadat u uw heup gebroken heeft, is in nauw overleg met de chirurg en klinisch geriater besloten of u geopereerd zou worden of niet.
Om inzicht te krijgen of patiënten ook achteraf tevreden zijn met die oorspronkelijke keuze, willen we u vragen om onderstaande vragen in te vullen.

Indien u **wel** geopereerd bent: Hoe tevreden bent u, op een schaal van 0 to 10, dat u in overleg met de behandelaars besloten heeft om geopereerd te worden?

Indien u **niet** geopereerd bent: Hoe tevreden bent u, op een schaal van 0 to 10, dat u in overleg met de behandelaars besloten heeft om niet geopereerd te worden?

(Graag één antwoord aankruisen; 0 = uiterst ontevreden en 10 = uiterst tevreden)

		**Uiterst Ontevreden**									**Uiterst tevreden**	
		0	1	2	3	4	5	6	7	8	9	10	N.v.t.
1.	Tevredenheid	☐	☐	☐	☐	☐	☐	☐	☐	☐	☐	☐	☐*

* N.v.t. aankruisen indien de naaste deze vraag niet zelf beantwoordt.

De behandelend artsen hebben u (en uw naaste) informatie gegeven over de voordelen en nadelen van een behandeling **met** operatie en van een behandeling **zonder** operatie. Wij willen graag weten wat u van die informatie vond.

2. Wat vond u van de <u>hoeveelheid</u> informatie?
   - ☐ Veel teveel
   - ☐ Een beetje teveel
   - ☐ Precies genoeg
   - ☐ Een beetje te weinig
   - ☐ Veel te weinig

3. Was u tevreden met de <u>inhoud</u> van de informatie?
   - ☐ Ja, alles wat ik wilde weten is besproken
   - ☐ Nee, ik had graag meer willen weten over: ____________________

4. Vindt u dat bij de keuze van de behandeling voldoende is geluisterd naar wat voor u belangrijk was?
   - ☐ Ja, de artsen hebben samen met mij de beste behandeling gekozen
   - ☐ Nee, er was niet voldoende aandacht voor wat voor mij belangrijk was.

   Als u "nee" hebt aangekruist, kunt u dat toelichten? ____________________________

Versie 1.0, d.d. 11 april 2023 Pagina 1 van 1

ID: PNOM-Implementatie - ____ - ____ **Datum (dd-mm-jjjj): ___ - ___ - 20___**

Introductie:

- Deelnemer(s) welkom heten en bedanken voor hun deelname.
- Doel van het interview toelichten.
- Benoemen dat deelname betekent dat de antwoorden gebruikt zullen worden in het onderzoek. Dit zal te allen tijden anoniem zijn. Als de gesprekspartner dat niet wil, wordt het gesprek beëindigd.

Tevredenheid van naaste met de gekozen behandeling

Nadat uw naaste zijn/haar heup gebroken heeft, is in nauw overleg met de chirurg en klinisch geriater besloten of uw naaste geopereerd zou worden of niet.
Om inzicht te krijgen of patiënten en/of hun naasten ook achteraf tevreden zijn met die oorspronkelijke keuze, willen we u vragen om onderstaande vragen in te vullen.

Indien uw naaste **wel** geopereerd is: Hoe tevreden bent u, op een schaal van 0 to 10, dat in overleg met de behandelaars besloten was dat uw naaste geopereerd zou worden?

Indien uw naaste **niet** geopereerd is: Hoe tevreden bent u, op een schaal van 0 to 10, dat in overleg met de behandelaars besloten was dat uw naaste niet geopereerd zou worden?

(Graag één antwoord aankruisen; 0 = uiterst ontevreden en 10 = uiterst tevreden)

	**Uiterst Ontevreden** 0	1	2	3	4	5	6	7	8	9	**Uiterst tevreden** 10	N.v.t.
1. Tevredenheid	☐	☐	☐	☐	☐	☐	☐	☐	☐	☐	☐	☐*

* N.v.t. aankruisen indien de patiënt deze vraag zelf kan beantwoorden.

De behandelend artsen hebben u informatie gegeven over de voordelen en nadelen van een behandeling **met** operatie en van een behandeling **zonder** operatie. Wij willen graag weten wat u van die informatie vond.

2. Wat vond u van de hoeveelheid informatie?
   - ☐ Veel teveel
   - ☐ Een beetje teveel
   - ☐ Precies genoeg
   - ☐ Een beetje te weinig
   - ☐ Veel te weinig

3. Was u tevreden met de inhoud van de informatie?
   - ☐ Ja, alles wat ik wilde weten is besproken
   - ☐ Nee, ik had graag meer willen weten over:

   ____

4. Vindt u dat bij de keuze van de behandeling voldoende is geluisterd naar wat voor uw naaste belangrijk was?
   Als u "nee" hebt aangekruist, kunt u dat toelichten?
   - ☐ Ja, de artsen hebben samen met mij de beste behandeling voor mijn naaste gekozen
   - ☐ Nee, er was niet voldoende aandacht voor wat voor mijn naaste belangrijk was.

   ____

Versie 1.0, d.d. 11 april 2023 Pagina 1 van 1
