# Supplementary material for: Shared decision-making for non-operative management versus operative management of hip fractures in selected frail older adults with a limited life expectancy: a protocol for a nationwide implementation study
Source: BMJ Open. 2024 Apr 17;14(4):e083429. doi: 10.1136/bmjopen-2023-083429 (PMC11029367; doi:10.1136/bmjopen-2023-083429)
Supplement: Supplementary data [file bmjopen-2023-083429supp003.pdf]

ID: PNOM-Implementatie - ____ - ____ Datum (dd-mm-jjjj): ___ - ___ - 20___

Aanmeldformulier en contactgegevens nieuwe patiënt

A. Gegevens patiënt

1. Naam: ____________________
2. Adres: ____________________
3. Postcode + woonplaats: ____________________
4. Tel. nummer: ____________________
5. Mobiel nummer: ____________________
6. E-mail: ____________________
7. Includerend ziekenhuis: ____________________
8. PID: ____________________

B. Gegevens alternatieve contactpersoon/wettelijk vertegenwoordiger

1. Naam: ____________________
2. Relatie t.o.v. patiënt: ____________________
3. Adres: ____________________
4. Postcode + woonplaats: ____________________
5. Tel. nummer: ____________________
6. Mobiel nummer: ____________________
7. E-mail: ____________________

C. Akkoord om gegevens met onderzoekers te delen voor belafspraak over 4-6 weken

1. Wie ondertekent voor akkoord? ☐ Patiënt ☐ Wettelijk vertegenwoordiger
2. Datum _____ - _____ - _____ (dd-mm-yyyy)
3. Handtekening ____________________

Zeelenberg ML, *et al. BMJ Open* 2024; 14:e083429. doi: 10.1136/bmjopen-2023-083429
