# Supplementary material for: Shared decision-making for non-operative management versus operative management of hip fractures in selected frail older adults with a limited life expectancy: a protocol for a nationwide implementation study
Source: BMJ Open. 2024 Apr 17;14(4):e083429. doi: 10.1136/bmjopen-2023-083429 (PMC11029367; doi:10.1136/bmjopen-2023-083429)
Supplement: Author's manuscript [file bmjopen-2023-083429.draft_revisions.pdf]

BMJ Open is committed to open peer review. As part of this commitment we make the peer review history of every article we publish publicly available.

When an article is published we post the peer reviewers' comments and the authors' responses online. We also post the versions of the paper that were used during peer review. These are the versions that the peer review comments apply to.

The versions of the paper that follow are the versions that were submitted during the peer review process. They are not the versions of record or the final published versions. They should not be cited or distributed as the published version of this manuscript.

BMJ Open is an open access journal and the full, final, typeset and author-corrected version of record of the manuscript is available on our site with no access controls, subscription charges or pay-per-view fees (http://bmjopen.bmj.com).

If you have any questions on BMJ Open's open peer review process please email info.bmjopen@bmj.com

BMJ Open

Shared decision making for Palliative, NonOperative Management (PNOM) versus operative treatment of hip fractures in frail older adults with a limited life expectancy; a protocol for a nationwide implementation study

Journal:	*BMJ Open*
Manuscript ID	bmjopen-2023-083429
Article Type:	Protocol
Date Submitted by the Author:	20-Dec-2023
Complete List of Authors:	Zeelenberg, Miliaan; Erasmus MC, Trauma research unit, Dept Surgery<br>Oosterwijk, Paulieke; Sint Antonius Ziekenhuis, Department of Trauma Surgery<br>Willems, Hanna; University of Amsterdam, Department of Geriatrics<br>Gosens, Taco; Elisabeth-TweeSteden Ziekenhuis, Department of Orthopaedic Surgery<br>Den Hartog, D; Erasmus MC, Department of Trauma Surgery<br>Joosse, Pieter ; Noordwest Ziekenhuisgroep, Department of Trauma Surgery<br>Loggers, Sverre; Noordwest Ziekenhuisgroep, Department of Trauma Surgery<br>Nijdam, Thomas ; Sint Antonius Ziekenhuis, Department of Surgery<br>Pel-Littel, Ruth; Vilans<br>Polinder, Suzanne; Erasmus MC, Department of Public Health<br>Jan Schuijt, Henk ; Sint Antonius Hospital, Department of Trauma Surgery<br>Wijnen, Hugo; Rijnstate, Department of Clinical geriatrics<br>Van der Velde, Detlef; Sint Antonius Ziekenhuis, Department of Surgery<br>Van Lieshout, Esther M.M.; Erasmus MC, Trauma Research Unit dept of Surgery<br>Verhofstad, Michael; Erasmus MC, Trauma Surgery<br>, PNOM-Implementation Study Group; PNOM-Implementation study group
Keywords:	Frailty, Hip < ORTHOPAEDIC & TRAUMA SURGERY, Implementation Science, ORTHOPAEDIC & TRAUMA SURGERY, GERIATRIC MEDICINE

SCHOLARONE™
Manuscripts

Shared decision making for Palliative, NonOperative Management (PNOM) versus operative treatment of hip fractures in frail older adults with a limited life expectancy; a protocol for a nationwide implementation study

Miliaan L. Zeelenberg MD[1,*], Paulieke C. Oosterwijk MD[2,*], Hanna C. Willems MD PhD[3], Taco Gosens MD PhD[4], Dennis Den Hartog MD PhD[1], Pieter Joosse MD PhD[5], Sverre A.I. Loggers MD[1,5], Thomas M.P. Nijdam MD[2], Ruth E. Pel-Littel MD PhD[6], Suzanne Polinder PhD[7], Henk Jan Schuijt MD PhD[2], Hugo H. Wijnen MD[8], Detlef Van der Velde MD PhD[2], Esther M.M. Van Lieshout PhD MSc[1], Michael H.J. Verhofstad MD PhD[1], PNOM-Implementation study group#

[1]Trauma Research Unit Department of Surgery, Erasmus MC, University Medical Center Rotterdam, Rotterdam, The Netherlands

[2]Department of Trauma surgery, St. Antonius Hospital, Utrecht, The Netherlands

[3]Department of Internal Medicine and Geriatrics, Amsterdam University Medical Center, Amsterdam, The Netherlands

[4]Department of Orthopedics, Elisabeth Hospital (ETZ), Tilburg, The Netherlands

[5]Department of Surgery, Noordwest Hospital group, Alkmaar, The Netherlands

[6]Vilans, Center of Expertise for Long-Term Care, Utrecht, The Netherlands.

[7]Department of Public Health, Erasmus MC, University Medical Center Rotterdam, Rotterdam, the Netherlands

[8]Department of Clinical Geriatrics, Rijnstate Hospital, Arnhem, The Netherlands

For peer review only

For peer review only - http://bmjopen.bmj.com/site/about/guidelines.xhtml

PNOM-Implementation study group is listed in Online supplementary materials 1.

Corresponding author

Dr. E.M.M. Van Lieshout

Trauma Research Unit Department of Surgery

Erasmus MC, University Medical Center Rotterdam

P.O. Box 2040

3000 CA Rotterdam

The Netherlands

Phone: +31.10.7031050

Mail: e.vanlieshout@erasmusmc.nl

Author contributions

All main authors were involved in conceptualization and/or designing of the study, and the writing and revising of associated documents. MZ and PO wrote the draft of the main manuscript. EvL and HJS provided direct supervision. All main authors actively contributed to critical revision of the manuscript in multiple revision rounds. The PNOM-Implementation study group contains the representatives from each of the 14 participating hospitals. All authors have read and approved the final version of this manuscript.

Word count: 3318 words

For peer review only - http://bmjopen.bmj.com/site/about/guidelines.xhtml

Abstract

Background and purpose: Recent research has highlighted palliative, nonoperative management (PNOM) as a viable alternative for frail older adults with hip fractures in the final phase of life. This study aims to guide Dutch physicians and hospitals nationwide in a standardized implementation of shared decision making (SDM) regarding surgery or PNOM in selected frail older adults with a hip fracture.

Methods and analysis: The patient population for implementation includes frail older adults aged ≥70 years with an acute proximal femoral fracture, nursing home care or a similar level of care elsewhere, and at least one additional criterion (i.e. malnutrition, severe mobility impairment, or ASA ≥4). The two-year implementation study will be conducted in four phases. In phases 1 and 2, barriers and facilitators for implementation will be identified and an implementation protocol, educational materials, and patient information will be developed. Phase 3 will involve an implementation pilot in 14 hospitals across the Netherlands. The protocol and educational material will be improved based on healthcare provider and patient experiences gathered through interviews. Phase 4 will focus on upscaling to nationwide implementation and the effect of the implementation on PNOM rate will be measured using data from the Dutch Hip Fracture Audit.

Ethics and dissemination: The study was exempted by the local Medical Research Ethics Committee (MEC-2023-0270, 10-05-2023) and Medical Ethics Committee United (W23.083, 26-04-2023). The study's results will be submitted to an open access international peer-reviewed journal. It's protocols, tools and results will be presented at several national and international academic conferences of relevant orthogeriactric (scientific) associations.

For peer review only - http://bmjopen.bmj.com/site/about/guidelines.xhtml

Trial registration number: NCT06079905 at ClinicalTrial.gov (11-10-2023)

For peer review only

For peer review only - http://bmjopen.bmj.com/site/about/guidelines.xhtml

Article summary

Strengths and limitations of this study

- This study builds on an existing foundation of recent well-received (inter)national publications, countrywide initiatives involving shared-decision making, and recent demands for assistance in the implementation of palliative, nonoperative management in frail older adults with hip fractures.
- The study will employ a staged approach in four phases, involving implementation strategies at the individual, local, and national level, that range from local educational sessions to nationwide distribution at conferences and proposed guideline changes.
- The study design allows for regular adjustment of its implementation protocols and educational materials based on patient and health care provider feedback from interviews from 14 different hospitals throughout the Netherlands.
- While the study has an ambitious 2-year time scheme to achieve nationwide implementation in the hospital setting, it will provide an important foundation for further initiatives.

For peer review only

For peer review only - http://bmjopen.bmj.com/site/about/guidelines.xhtml

Introduction

A hip fracture in a frail older adults can indicate an approaching end of life. Advanced age, multiple comorbidities, and severe mobility limitations are important risk factors for early post-operative mortality after a hip fracture (1, 2). Studies show that >30% of patients die within one year post hip fracture, with six-month mortality rates in older adults with dementia exceeding 55%, often with low quality of life in the months preceding death (3-5). These patients, in their final phase of life, often do not benefit from an operative treatment that aims to restore mobility. Based on this insight, a paradigm shift is occurring in clinical approach, emphasizing the importance of individualized care plans focused on optimal quality of life (6, 7).

The FRAIL-HIP study has confirmed that palliative nonoperative management (PNOM) can be a good alternative to surgical management in select frail older adults (5, 8-10). Quality of life after PNOM was valued non-inferiorly and the majority of the next of kin rated the quality of dying of their relative as very good (5, 8). Despite higher mortality rates in patients opting for PNOM, the option to allow patients to pass away in a familiar environment was a compelling factor in favor of PNOM (11). Following these results, multiple Dutch hospitals have asked for guidance on implementing PNOM in their institutions.

Central to PNOM is the shared decision making (SDM) process, involving patients and their families in treatment decisions aligned with the patients' preferences and needs. Based on the results of the FRAIL-HIP study and other literature, an implementation protocol has been drafted, piloted, and refined based on experiences of healthcare professionals (7, 9, 12). This protocol version will serve as the starting point for the iterative process towards nationwide implementation.

For peer review only - http://bmjopen.bmj.com/site/about/guidelines.xhtml

The aim of this study is nationwide implementation of SDM on treatment of hip fractures, involving both PNOM and operative treatment, to ensure that frail older adults receive optimal, patient-centered care in the final phase of their lives.

For peer review only

For peer review only - http://bmjopen.bmj.com/site/about/guidelines.xhtml

Methods and analysis

Study design and aims

This two-year implementation study will consist of four phases (Figure 1); development of the implementation protocol and educational material (Phase 1 & 2), implementation across a subset of hospitals across the Netherlands and process evaluation and optimization of the protocol based on healthcare provider/patient experiences (Phase 3), and upscaling towards nationwide implementation and project evaluation (Phase 4). The study design of phases 3 and 4 entails a multicenter prospective cohort study. Overall, the project will cover all activities as recommended in the ImpRes Guide, which mentions four implementation phases (*i.e.*, exploration, installation, initial implementation, and full implementation) and should efficiently translate local knowledge into general knowledge (13). The 'Consolidated Framework for Implementation Research' (CFIR) was used for designing this project (14, 15). With education being a critical part of the implementation, elements of the education strategy from Wensing *et al.* (16) were added. The CFIR domains that the four phases of this project will cover are mentioned in Table 1.

To achieve the primary aim, this study will use a staged approach in four phases that involves three levels: The healthcare provider specific/local hospital level (micro) in phases 1-3, the level of a limited number of hospitals (meso) in phase 3, and the national level (macro) in phase 4. The four consecutive phases of the project have phase-specific aims with corresponding target populations and points of action, that are described in Table 2. To ensure nationwide implementation many parties and interest groups are or will be involved in the project (Table 3).

For peer review only - http://bmjopen.bmj.com/site/about/guidelines.xhtml

Study population

Phases 1 and 2 will not include patients or patient data but will include healthcare provider and patient association perspectives. Phases 3 and 4 will also include frail older adults with a limited life expectancy who sustained a proximal femoral fracture. Patients will be included and receive SDM on PNOM and operation if they meet the following criteria:

1. Acute proximal femoral fracture (AO type 31), confirmed on X-ray or CT-scan
2. 70 years or older
3. Living in a nursing home or receiving similar care at home
4. And at least one of the following characteristics:
    - Malnutrition (cachexia or BMI <18.5 kg/m2)
    - Functional ambulation category* (FAC) 2 or lower
    - Severe comorbidities (ASA category 4 or 5)

Patients and/or proxies that do not have sufficient comprehension of the Dutch language will be excluded from the interviews during phase 3.

*The Functional Ambulation Categories (FAC) is a functional walking test that evaluates ambulation ability (17). This 6-point scale assesses ambulation status by determining how much human support the patient requires when walking, regardless of whether or not they use a personal assistive device. FAC ≤2 means that a patients at least has the need for (intermittent) help of another person to be able to ambulate (FAC 2) to no functional ambulatory capabilities (FAC 0).

Implementation in four phases

For peer review only - http://bmjopen.bmj.com/site/about/guidelines.xhtml

Phase 1 (exploration): evaluate current practice and identify facilitators and barriers of PNOM implementation

Based on the evidence-based 'Measurement Instrument for Determinants of Innovations' (MIDI) (18, 19) and the 'Barriers and Facilitators Assessment Instrument' (BFAI) (20) a survey has been developed, aimed to identify the facilitators and barriers for applying SDM and PNOM for selecting the best treatment for individual patients of the target population and identified five barriers and 23 facilitators (9). The patient perspective was not included in this study, but was explored in another study (11). A panel from older adults organizations, general practitioners and geriatric specialists will discuss and supplement the data from the aforementioned studies from their perspective.

Phase 2 (installation): develop an implementation strategy for PNOM

A draft protocol for PNOM implementation, with as focal point the SDM interview, has been written based on the experience and expertise of the project members, previous studies, and questionnaires. This protocol contains the central vision, the requirements for the implementation of PNOM, and a recommendation for the composition of a local project team of stakeholders and drivers of change. To remove barriers, practical tools such as pocket cards, posters, factsheets, and local training options will be drafted or updated by the project team, based on the 'Expert Recommendations for Implementing Change strategies' (21). This should help drivers of change in achieving implementation goals and change day-to-day policies. The PNOM protocol will contain an overview of the data to be collected for PNOM evaluation. A reporting tool will be developed in the Dutch Hip Fracture Audit (DHFA) for this purpose. In addition, educational modules for healthcare professionals will be developed using the PNOM protocol in combination

For peer review only - http://bmjopen.bmj.com/site/about/guidelines.xhtml

with existing educational modules on SDM (22-24). PNOM as a treatment option will also be included in the obligatory curriculum for residents by the Association of Surgeons of the Netherlands (in Dutch: Nederlandse Vereniging voor Heelkunde, NVvH), in order to incorporate PNOM into the educational program of (orthopedic) trauma residents.

PHASE 3 (initial implementation): PNOM-implementation in selected hospitals

PNOM will be implemented in 14 selected hospitals (Figure 2, collaborators listed in Online Resource 1). Based on the experience of these hospitals, the PNOM implementation protocol and/or the educational material will be optimized. To safeguard implementation of PNOM in the long term, the PNOM protocol pays explicit attention to the involvement of all relevant healthcare providers by informing those involved before, during and after implementation, and by collecting data on relevant feasibility parameters as described in the Bowen model (25). To achieve this, the site principal investigator will be asked to establish a local project team. The team should represent all groups involved in PNOM, including relevant physicians and, as applicable, residents of the involved departments, specialized nurses, physician assistants, and hospital management. Prior to implementation, this local team will meet with the researchers for an interview to train them in applying the SDM and the PNOM implementation protocol, and to collect details on their specific needs for the implementation.

The local team will perform patient selection and apply SDM on PNOM and operative treatment for all patients who meet the eligibility criteria. After the start of the implementation, during the first six months, patients, relatives, and/or next of kin will be approached once by telephone to ask about their satisfaction with the treatment decision process, and the chosen treatment. This will be done through a semi-structured interview. To provide more insight about

For peer review only - http://bmjopen.bmj.com/site/about/guidelines.xhtml

their own patient population the interview results will be discussed with the participating centers on a quarterly basis. All collected data will be used to improve the PNOM protocol and/or educational material prior to phase 4. Six months after start of implementation, the local team will be approached again for an interview to evaluate the process with the relevant parameters: adoption, integration, applicability, expansion, and early local effectiveness.

PHASE 4 (nationwide implementation): PNOM-implementation in all hospitals in the Netherlands and project analysis

Phase 4 is an expansion of phase 3 to reach nationwide implementation. The implementation strategy will essentially be the same as in phase 3. Due to the increase in number of hospitals, the supervision and contact with the researchers and steering committee will be less intensive than in phase 3. In practice, this means that the PNOM protocol will be implemented, but the interviews with health care professionals in the hospitals (prior to start and six months after start) will be omitted. To ensure that all information on SDM and PNOM implementation is available continuously to both patients and healthcare providers, a dedicated website will be developed and maintained. To effectively reach a broad audience of healthcare providers and keep them updated on the implementation process and learning experiences, our goal is to give presentations at the annual meetings of orthogeriatric scientific associations, as well as associations for general practitioners and elderly care physicians (Table 3). In addition, time slots for presentations will be requested at the meetings of the regional trauma networks. Ultimately, we aim to include SDM on PNOM or operative treatment for proximal femoral fractures in select frail older adults in the National guidelines for hip fracture treatment and operative care in older adults.

For peer review only - http://bmjopen.bmj.com/site/about/guidelines.xhtml

Outcome measures and data collection

The primary outcome measure (for phase 3 and 4) is (change in) the PNOM rate. Type of treatment (PNOM versus operative treatment) will be extracted from the Dutch Hip Fracture Audit (DHFA). The DHFA is a national database, including over 95% of Hip fracture patients, and can provide automated anonymous patient data. To evaluate the effect of the implementation, the change in PNOM rate for the targeted population will be evaluated and compared with the rate prior to implementation. The secondary outcome measures (for phase 3 only) will be collected during semi-structured interviews with patients/proxy or next of kin. These are the satisfaction with the chosen treatment on a numeric rating scale (0-10), their valuation of the information received during the SDM process, and if the patient's needs were respected enough in the treatment decision.

The following patient data will be also collected in phases 3 and 4 from the DHFA (emerging from the patients' medical records). Patient characteristics (age, sex, ASA, Functional Ambulation Category), pre-fracture situation (residence, mobility, KATZ Activities of Daily Living (ADL) score), fracture details (fracture classification, affected side), hospital details (hospital length of stay and intensive care length of stay), and outcome details (complications, revision surgeries, readmission, 30-day mortality, and time until death).

Data will be collected for each patient who meets the eligibility criteria. To reduce the administrative burden, the DHFA database will be used as much as possible. Data from the DHFA will be imported in a Castor EDC database, in which hospitals can only see their own data. The datasets of all hospitals will be combined for the final analysis.

For peer review only - http://bmjopen.bmj.com/site/about/guidelines.xhtml

Sample size calculation

A formal sample size calculation was not made as the primary aim is implementation on a national level. To give an idea of the total national population size the following estimate was made: In 2020 21,165 patients presented to a hospital in the Netherlands with a proximal femoral fracture (26). Of these 17,225 (81.5%) were aged 70 years or older. An approximate 25% of this population lived in a nursing home or other institution. Which projects to about 4,300 patients as the primary target population of this study. Approximately half of these patients will meet our inclusion criteria and thus not all patients will be eligible for PNOM, but this gives a broad estimate of the annual number of patients that should at least be considered for SDM on PNOM and operative treatment.

Statistical analysis

Data will be analyzed using the Statistical Package for the Social Sciences (SPSS) version 28 or higher (SPSS, Chicago, Ill., USA). Normality of continuous data will be tested with the Shapiro-Wilk test, and homogeneity of variances will be tested using the Levene's test. A p-value <0.05 will be taken as threshold of statistical significance. Missing values will not be imputed.

The primary outcome, PNOM rate, will be determined by dividing the number of patients treated with PNOM by the total eligible number of patients (per participating center and for all participating centers combined).

All continuous data will be reported as mean with standard deviation (SD; if normally distributed based on Shapiro-Wilk test) or as median with quartiles (if not normally distributed). Categorical data will be reported as number with percentage. Pre- and post-implementation data will be compared with a Student T-test (continuous data, normally distributed), Mann-Whitney

For peer review only - http://bmjopen.bmj.com/site/about/guidelines.xhtml

U-test (continuous data, non-normally distributed), or a Chi-square or Fisher's Exact test (categorical data), as applicable. The analysis will be stratified by PNOM versus surgery, and further stratified as feasible depending on the final study population, e.g., by time since PNOM implementation, pre-fracture mobility, ADL status, fracture classification, or bilateral fracture.

For peer review only

For peer review only - http://bmjopen.bmj.com/site/about/guidelines.xhtml

Ethics and dissemination

Ethical approval, risks and dissemination

The study was exempted by the local Medical Research Ethics Committee (MEC-2023-0270, 10-05-2023) and Medical Ethics Committee United (W23.083, 26-04-2023) and is registered at ClinicalTrial.gov (NCT06079905, 11-10-2023). The study was approved by the board of directors of all hospitals participating in phase 3. There are no risks involved in this study. The only burden is the time needed for the interviews with health care providers and patients/next of kin in phase 3. The interviews will be performed by researchers following an opt-out design, participants can stop at any time. Participants will be asked to consent to share their contact details with the researchers and participation means interview data can be used. Besides this, only already existing and routinely collected data will be collected. All treatments, chosen after SDM, will be performed following local standard care protocols. Based on the above, the overall burden and risks are considered negligible. The study will follow Good Clinical Practice (GCP) guidelines. The study's results will be submitted to an open access international peer-reviewed journal. As previously explained above, it's protocols, tools and results will be made publicly available and presented at several national and international academic conferences of relevant orthogeriactric (scientific) associations.

Data management, monitoring, and privacy

All data and information collected regarding this study will be treated confidentially by the researchers. Research data that can be traced to individual persons can only be viewed by authorized personnel (members of the research team and members of the health care inspection).

For peer review only - http://bmjopen.bmj.com/site/about/guidelines.xhtml

Data will be encoded and stored in a Castor EDC database with password restricted access to the researchers at individual sites only. Regarding participants privacy, the Dutch 'General data protection regulation (GDPR) implementation act' will be applied.

Patient and public involvement

A patient association representative was consulted during the drafting of the study protocol and could provide input for the study design and implementation tools. Through interviews, study participants (patients, next-of-kin and healthcare providers) will be directly involved in providing feedback on the SDM process and educational tools used during the project. In addition to general publication, a summary of the main results will be made available to study participants on request.

Funding and disclosures

This project has been funded by the Netherlands Organization for Health Research and Development (ZonMw; project No. 10390162210008). The funder of the study has no role in study design, data collection, data analysis, data interpretation, or writing of the manuscript. All authors declare no conflicts of interest or competing interests for this project.

For peer review only

For peer review only - http://bmjopen.bmj.com/site/about/guidelines.xhtml

Discussion

Clinical impact

This project provides an important translation of recent scientific publications to clinical practice and its success could play an important role in (inter)national surgical and geriatric guideline changes. Successful implementation of PNOM will change the way healthcare providers view acute hip fractures in frail older adults. Instead of primarily focusing on clinical possibility of hip fracture treatment, the focus will shift towards treatment concerning patients' quality of life and harm reduction in the final stages of life. Because of this shift, the PNOM rate is expected to rise in this select group. However, the main goal is not to increase PNOM rate, but to provide each patient with the clinical care that best suits their wishes, needs, and clinical reality. Nationwide implementation of SDM on PNOM will also lead to a reduction of overtreatment and might reduce associated costs (10). Another important outcome is reduced practice variation between hospitals in both patient selection for (SDM on) PNOM and nationwide implementation of a universal structured SDM protocol for frail older adults with a hip fracture.

Strengths and limitations

The strength of this study is that it builds on an existing foundation of recent well-received (inter)national publications, countrywide initiatives involving SDM and advance care planning, and recent demands for assistance in the implementation of PNOM from several hospitals. The project group includes experienced professionals from (orthopedic-) trauma surgery, geriatrics, epidemiology, and implementation science from both university medical centers and general hospitals, supplemented with patient perspectives and experts in SDM and study methodology. A

For peer review only - http://bmjopen.bmj.com/site/about/guidelines.xhtml

limitation of the project is the limited time period for the initial project. Achieving nationwide implementation within two years, which is the maximum allowed project duration of the grantor, might be an overoptimistic aim. However, the project will have developed a strong foundation on which future projects can build and will keep sharing its tools and information, even after completion. Another limitation is that the inclusion criteria might prove to be too narrow. There might be a larger group of patients for whom PNOM could suit their preferences or goals of care. However, the project group has decided to keep strict criteria to prevent the pendulum swinging too far and causing undertreatment with long-term survival without functional recovery after opting for palliative, nonoperative treatment, which results in potential decreased quality of life and associated adverse events. Depending on the results of the initial implementation the inclusion criteria in the protocol might be adjusted to suit the needs of patients and health care providers. Finally, the PNOM rate in the DHFA database might be an underestimation, as patients living in a nursing home might already have advance care plans preventing them from being transferred to the hospital in the case of a hip fracture. As the PNOM implementation gains national traction more attention to advance care planning around hip fractures might lead to an unmeasured increase of the PNOM rate in the pre-hospital setting. While reducing DHFA registration for this project, improved communication on patients wishes and integration of hip fractures in advance care planning, between patients and (geriatric) medical practitioners within and outside the hospital, is a desired effect that will improve the quality of PNOM and patients' treatment satisfaction.

Future perspective

For peer review only - http://bmjopen.bmj.com/site/about/guidelines.xhtml

The success of this project can be used as an international example for trauma care in frail older adults and lead to further changes in advance care planning within this older trauma population. The projects finished protocols and clinical tools may be used for updating the Dutch national 'Guideline for the treatment of proximal femoral fractures' and 'Guideline for multidisciplinary treatment of operative treatment of frail older adults' and may also help updating international treatment guidelines on proximal femoral fractures in frail older adults.

For peer review only

For peer review only - http://bmjopen.bmj.com/site/about/guidelines.xhtml

References

1. Nijmeijer WS, Folbert EC, Vermeer M, Slaets JP, Hegeman JH. Prediction of early mortality following hip fracture surgery in frail elderly: The Almelo Hip Fracture Score (AHFS). Injury. 2016;47(10):2138-43.
2. Van de Ree CL, Gosens T, Van der Veen AH, Oosterbos CJ, Heymans MW, De Jongh MA. Development and validation of the Brabant Hip Fracture Score for 30-day and 1-year mortality. Hip Int. 2020;30(3):354-62.
3. Hu F, Jiang C, Shen J, Tang P, Wang Y. Preoperative predictors for mortality following hip fracture surgery: a systematic review and meta-analysis. Injury. 2012;43(6):676-85.
4. Morrison RS, Siu AL. Survival in end-stage dementia following acute illness. JAMA. 2000;284(1):47-52.
5. Loggers SAI, Van Balen R, Willems HC, Gosens T, Polinder S, Ponsen KJ, et al. The Quality of Dying in Frail Institutionalized Older Patients After Nonoperative and Operative Management of a Proximal Femoral Fracture: An In-Depth Analysis. Am J Hosp Palliat Care. 2023:10499091231180556.
6. Schuijt HJ, Lehmann LS, Javedan H, von Keudell AG, Weaver MJ. A Culture Change in Geriatric Traumatology: Holistic and Patient-Tailored Care for Frail Patients with Fractures. J Bone Joint Surg Am. 2021;103(18):e72.
7. Nijdam TMP, Laane D, Schiepers TEE, Smeeing DPJ, Kempen DHR, Willems HC, et al. The goals of care in acute setting for geriatric patients in case of a hip fracture. Eur J Trauma Emerg Surg. 2023;49(4):1835-44.
8. Loggers SAI, Willems HC, Van Balen R, Gosens T, Polinder S, Ponsen KJ, et al. Evaluation of Quality of Life After Nonoperative or Operative Management of Proximal Femoral Fractures in Frail Institutionalized Patients: The FRAIL-HIP Study. JAMA Surg. 2022;157(5):424-34.
9. Spronk I, Loggers SAI, Joosse P, Willems HC, Van Balen R, Gosens T, et al. Shared decision making for the treatment of proximal femoral fractures in frail institutionalized older patients: healthcare providers' perceived barriers and facilitators. Age Ageing (in print). 2022.

For peer review only - http://bmjopen.bmj.com/site/about/guidelines.xhtml

10. Loggers SAI, Geraerds A, Joosse P, Willems HC, Gosens T, Van Balen R, et al. Nonoperative versus operative management of frail institutionalized older patients with a proximal femoral fracture: a cost-utility analysis alongside a multicenter prospective cohort study. Osteoporos Int. 2023;34(3):515-25.

11. Nijdam TMP, Laane D, Spierings JF, Schuijt HJ, Smeeing DPJ, van der Velde D. Proxy-reported experiences of palliative, non-operative management of geriatric patients after a hip fracture: a qualitative study. BMJ Open. 2022;12(8):e063007.

12. Palliatief niet-operatief management bij kwetsbare oudere patiënten met een heupfractuur. Implementatiehandleiding Versie 1.1.1., d.d. 20-06-2022.

13. Center for Implementation Science Research. Implementation science research development (ImpRes) tool 2018 [Available from: https://impsci.tracs.unc.edu/wp-content/uploads/ImpRes-Guide.pdf. .

14. Damschroder LJ, Aron DC, Keith RE, Kirsh SR, Alexander JA, Lowery JC. Fostering implementation of health services research findings into practice: a consolidated framework for advancing implementation science. Implement Sci. 2009;4:50.

15. Birken SA, Powell BJ, Shea CM, Haines ER, Alexis Kirk M, Leeman J, et al. Criteria for selecting implementation science theories and frameworks: results from an international survey. Implement Sci. 2017;12(1):124.

16. Wensing M, Fluit C, Grimshaw J, Grol R. Educational Implementation Strategies. In Improving Patient Care (eds M. Wensing, R. Grol and J. Grimshaw. 2020.

17. Holden MK, Gill KM, Magliozzi MR, Nathan J, Piehl-Baker L. Clinical gait assessment in the neurologically impaired. Reliability and meaningfulness. Phys Ther. 1984;64(1):35-40.

18. Fleuren MA, Paulussen TG, Van Dommelen P, Van Buuren S. Towards a measurement instrument for determinants of innovations. Int J Qual Health Care. 2014;26(5):501-10.

19. Fleuren M, Paulussen T, Van Dommelen P, Van Buuren S. Measurement instrument for determinants of innovations (MIDI). Leiden: TNO. 2014.

20. Harmsen M, Peters M, Wensing M. Barriers and facilitators assessment instrument: Introduction, instructions and instrument. Nijmegen: IQ healthcare Scientific Institute for Quality of Healthcare. Radboud University Medical Center Nijmegen. 2005.

For peer review only - http://bmjopen.bmj.com/site/about/guidelines.xhtml

21. Powell BJ, Waltz TJ, Chinman MJ, Damschroder LJ, Smith JL, Matthieu MM, et al. A refined compilation of implementation strategies: results from the Expert Recommendations for Implementing Change (ERIC) project. Implement Sci. 2015;10:21.
22. Dialuge Trainer. Samen beslissen met ouderen met multimorbiditeit: Dialogue Trainer; [Available from: https://nl.dialoguetrainer.app/module/samenbeslissengeriatrie-open/.
23. FMS. Samen Beslissen (Shared decision making): Federatie Medisch Specialisten; 2023 [Available from: https://demedischspecialist.nl/themas/thema/samen-beslissen-0.
24. Kennisplatform Uitkomstgerichte Zorg. Samen beslissen: Kennisplatform Uitkomstgerichte Zorg; [Available from: https://www.platformuitkomstgerichtezorg.nl/themas/samen+beslissen/default.aspx.
25. Bowen DJ, Kreuter M, Spring B, Cofta-Woerpel L, Linnan L, Weiner D, et al. How we design feasibility studies. Am J Prev Med. 2009;36(5):452-7.
26. CBS. Hospital admissions and patient diagnosis (Ziekenhuisopnamen en -patiënten; diagnose-indeling): Centraal Bureau Statistiek (Dutch Central Bureau for Statistics) 2022 [updated 16-06-2022. Available from: https://opendata.cbs.nl/statline/#/CBS/nl/dataset/84069NED/table?ts=1670493690817.

For peer review only

For peer review only - http://bmjopen.bmj.com/site/about/guidelines.xhtml

Figures and Tables

Figure 1: Schematic overview of the implementation process in 4 phases

DHFA, Dutch Hip Fracture Audit; PNOM, palliative nonoperative management

Figure 2: Map of participating hospitals during the initial implementation in selected hospitals (Phase 3)

For peer review only

For peer review only - http://bmjopen.bmj.com/site/about/guidelines.xhtml

Table 1: CFIR domains and their role in the implementation project

CFIR Domain	Role in project
Inner setting	Gain insight into the hospital/implementation location, including internal network and communication, culture (norms and values), knowledge, current use of the intervention, and implementation climate (tension for change, feedback, learning climate)
Outer setting	Gain insight into the needs of patients/proxies and pressure from peers (need for hospitals to use the same protocol).
Characteristics of individuals	Gain insight into the confidence/preparedness to discuss and apply PNOM through SDM with the intended target group
Characteristics of the intervention	Gain insight into the SDM conversation as part of PNOM, the needs of healthcare providers on how to conduct SDM and who participates in it, and what they need to apply PNOM.

CFIR, Consolidated Framework for Implementation Research; PNOM, palliative nonoperative management; SDM, shared decision making

For peer review only

For peer review only - http://bmjopen.bmj.com/site/about/guidelines.xhtml

Table 2: Study aims with specific action points per targeted group for the four study phases

Points of action per phase	Targeted group	Aim/outcome
Phase 1		
Qualitative research on patient experiences and perceived barriers and facilitators for SDM on PNOM	Patients, next-of-kin and healthcare providers*	Enriching the implementation protocol with recent research on patient/healthcare provider experiences regarding SDM on PNOM versus operative treatment.<br>Providing insight into barriers and facilitators on the local and individual level
Phase 2		
Development of educational material on SDM with frail older adults with hip fractures for patients and healthcare providers<br>Implementing SDM on PNOM in the curriculum for (orthopedic) surgeons in training	Patients, next-of-kin and healthcare providers*	Increasing (practical) knowledge on performing SDM on PNOM in healthcare providers.<br>Providing healthcare providers with tools to prepare patients and next-of-kin for SDM on PNOM versus operative treatment
Phase 3		
Presentations of the Implementation protocol and included tools at annual meetings of the involved scientific associations	Healthcare providers*, members of associated scientific associations	Creating awareness and a sense of urgency for SDM implementation and identifying local drivers of change for future implementation
Implementation of the PNOM implementation protocol and evaluation through interviews with patients and healthcare providers in 14 selected Dutch hospitals	Local project teams and drivers of change in 14 Dutch hospitals and patients/next-of-kin	Evaluating and improving the implementation protocol and educational modules based on local experiences, barriers and facilitators through patient- and healthcare provider interviews
Phase 4		
Maintaining and improving awareness through conference- and regional trauma network presentations<br>Identification of local ambassadors/drivers of change<br>Dissemination of the implementation protocol/educational tools through a dedicated website, scientific publications and media coverage	Health care providers*, and specifically, local drivers of change, in all Dutch hospitals treating frail older adults with hip fractures	Upscaling of the implementation to all hospitals in the Netherlands that treat the targeted patient group
Measuring the effect of implementation, changes in PNOM rate, through the DHFA database	Dutch patients and hospitals through the DHFA database	Evaluating changes in practice after implementation

For peer review only - http://bmjopen.bmj.com/site/about/guidelines.xhtml

*Healthcare providers include (orthopedic) trauma surgeons, geriatricians, acute care physicians, anesthesiologists, residents, and physician assistants directly involved in (acute) care for frail older adults with a hip fracture in Dutch hospitals.

DHFA, Dutch Hip Fracture Audit; PNOM, palliative nonoperative management; SDM, shared decision making

For peer review only

For peer review only - http://bmjopen.bmj.com/site/about/guidelines.xhtml

Table 3: Participating parties and their roles and responsibilities

Participator(s)	Roles and responsibilities
Central project group/steering committee	The project group is responsible for the general oversight of the entire project.
Participating hospitals	They will implement the PNOM protocol locally, and will provide data needed to evaluate the effect of the implementation. They will need to allow local training, and participate in interviews to gain insight into their experience with PNOM implementation.
Patients and/or proxies	Will be asked to voluntarily participate in interviews and provide feedback on the chosen treatment and SDM process.
Dutch Hip Fracture Audit (DHFA) and Dutch Institute for Clinical Auditing (DICA)	They will provide the data so the hospitals can use it as mirror information. The DHFA data (which are existing data) will be used as pre and post implementation data, and will thus allow accurate monitoring of the impact of PNOM implementation.
Scientific associations (Dutch Trauma Society (NVT), Dutch Association of Surgeons of the Netherlands (NVvH), Dutch Orthopedic Association (NOV), and Dutch Association of Clinical Geriatrics (NVKG))	The scientific associations are responsible for education for medical specialists and residents-in-training. They will play a key role in the education sessions, and in forwarding information on our project to their members.
Members of the National Association of General Practitioners (LHV) and the Association for Older Adult Care Physicians (Verenso)	Will be kept informed about this project through mailings and presentations during their annual conferences.

DHFA, Dutch Hip Fracture Audit; DICA, Dutch Institute Clinical Auditing; LHV, Landelijke Huisartsen Vereniging; NVKG, Nederlandse Vereniging voor Klinische Geriatrie; NVT, Nederlandse Vereniging voor Traumachirurgie; NVvH, Nederlandse Vereniging voor Heelkunde; NOV, Nederlandse Vereniging voor Orthopedie; PNOM, palliative nonoperative management; SDM, shared decision making;

For peer review only

For peer review only - http://bmjopen.bmj.com/site/about/guidelines.xhtml

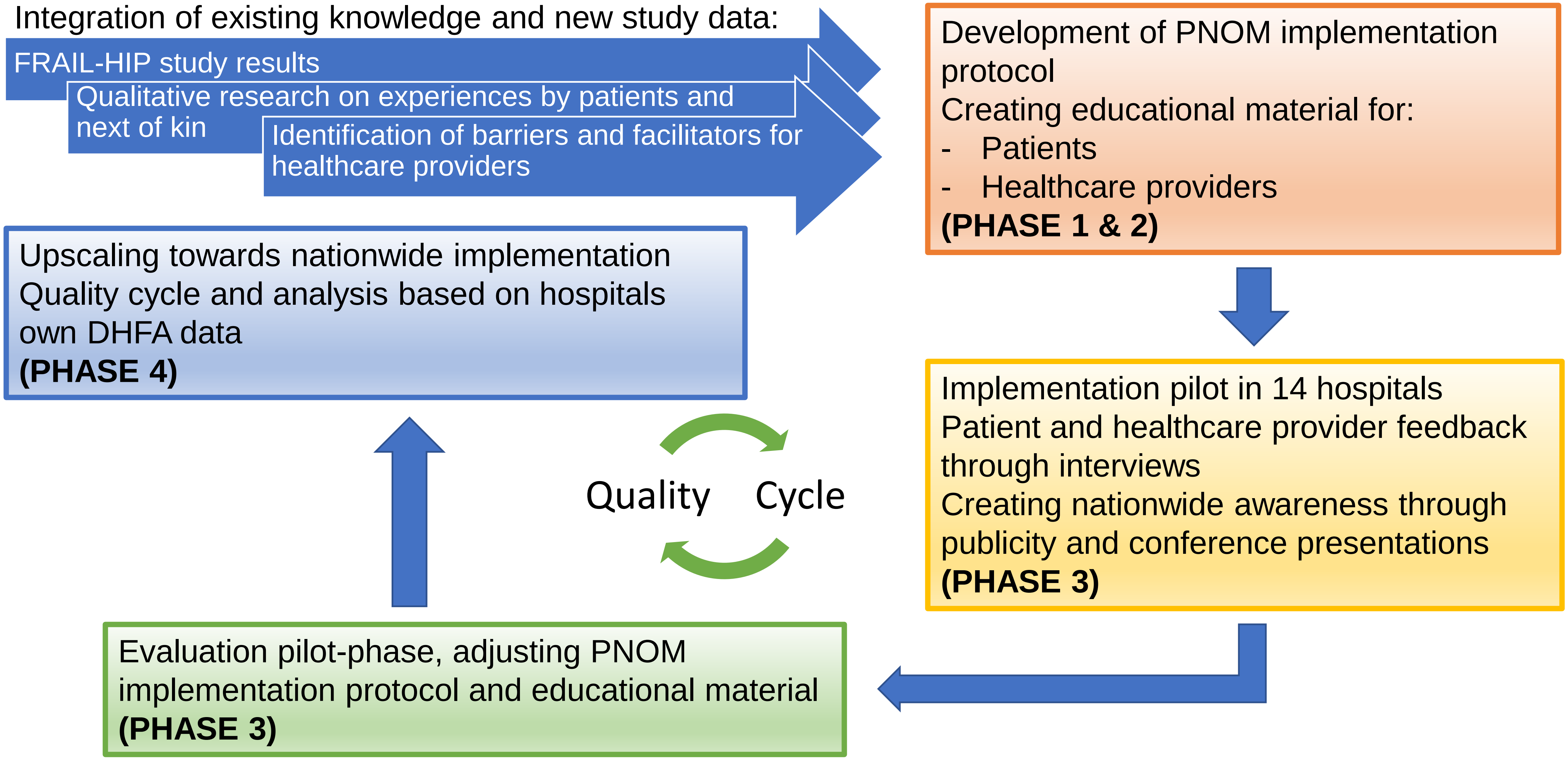
Integration of existing knowledge and new study data:
FRAIL-HIP study results
Qualitative research on experiences by patients and next of kin
Identification of barriers and facilitators for healthcare providers
Development of PNOM implementation protocol
Creating educational material for:
- Patients
- Healthcare providers
(PHASE 1 & 2)
Implementation pilot in 14 hospitals
Patient and healthcare provider feedback through interviews
Creating nationwide awareness through publicity and conference presentations
(PHASE 3)
Evaluation pilot-phase, adjusting PNOM implementation protocol and educational material
(PHASE 3)
Quality Cycle
Upscaling towards nationwide implementation
Quality cycle and analysis based on hospitals own DHFA data
(PHASE 4)

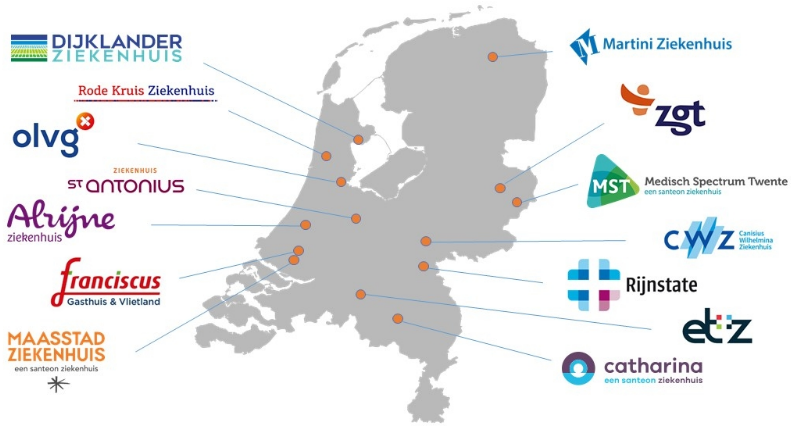

Figure 2: Map of participating hospitals during the initial implementation in selected hospitals (Phase 3)

338x190mm (96 x 96 DPI)

Online supplementary materials 1

Table S1: Contributors in PNOM-Implementation study group

Institution	Location	First name	Last name	Title/Academic degree	Medical specialty
Alrijne ziekenhuis	Leiderdorp	E.	Sohl	MD PhD	Geriatrics
Alrijne ziekenhuis	Leiderdorp	J.A.	Jansen	MD	Orthopedics
Alrijne ziekenhuis	Leiderdorp	M.	Leijnen	MD PhD	Surgery
Carnisius Wilhelmina ziekenhuis	Nijmegen	W.A.H.	Van der Stappen	MD	Surgery
Carnisius Wilhelmina ziekenhuis	Nijmegen	J.	Jansen	MD PhD	Orthopedics
Carnisius Wilhelmina ziekenhuis	Nijmegen	M.	Peters-Kop	PA	Geriatric trauma unit
Catharina ziekenhuis	Eindhoven	A.H.	Van der Veen	MD PhD	Surgery
Catharina ziekenhuis	Eindhoven	N.C.	Schepel	MD	Orthopedics
Catharina ziekenhuis	Eindhoven	J.A.M.	Wilmer	MD	Geriatrics
Dijklander ziekenhuis	Hoorn	J.	Steens	MD PhD	Orthopedics
Dijklander ziekenhuis	Hoorn	J.	Winkelhagen	MD	Surgery
Dijklander ziekenhuis	Hoorn	N.M.F.	Noorda	MD	Geriatrics
Elisabeth TweeSteden ziekenhuis	Tilburg	H.A.A.M.	Maas	MD PhD	Geriatrics
Franciscus ziekenhuis	Rotterdam	O.	Wijers	MD PhD	Surgery
Franciscus ziekenhuis	Rotterdam	V.	Vis	PA	Geriatric trauma unit
Maasstad ziekenhuis	Rotterdam	D.	Van der Stap	MD	Geriatrics
Maasstad ziekenhuis	Rotterdam	G.R.	Roukema	MD PhD	Surgery
Martini ziekenhuis	Groningen	E.	Bosma	MD	Surgery
Martini ziekenhuis	Groningen	T.M.	Van Raaij	MD PhD	Orthopedics
Martini ziekenhuis	Groningen	A.M.	Van der Knaap	MD	Geriatrics
Medisch spectrum Twente	Enschede	R.	de Groot	MD PhD	Surgery
Medisch spectrum Twente	Enschede	A.V.C.M.	Zeegers	MD PhD	Orthopedics
OLVG	Amsterdam	H.A.	Formijne Jonkers	MD PhD	Surgery
OLVG	Amsterdam	D.H.R.	Kempen	MD PhD	Orthopedics
OLVG	Amsterdam	K.	De Vries	MD	Geriatrics
Rijnstate ziekenhuis	Arnhem	A.F.	Pull ter Gunne	MD PhD	Surgery
Rijnstate ziekenhuis	Arnhem	M.P.	Somford	MD PhD	Orthopedics
Rode Kruis ziekenhuis	Beverwijk	E.	Tanis	MD PhD	Surgery
Rode Kruis ziekenhuis	Beverwijk	J.H.	Duits	MD	Geriatrics
Rode Kruis ziekenhuis	Beverwijk	R.A.	Zandbergen	MD	Orthopedics
St. Antonius ziekenhuis	Nieuwegein	N.C.	Leegwater	MD PhD	Orthopedics

For peer review only

St. Antonius ziekenhuis	Nieuwegein	O.C.	Geraghty	MD PhD	Geriatrics
Ziekenhuisgroep Twente	Almelo	J.H.	Hegeman	MD PhD	Surgery
Ziekenhuisgroep Twente	Almelo	E.M.	Regtuijt	MD	Geriatrics

MD, Doctor of medicine; PA, Physician assistant; PhD, Doctor of philosophy

For peer review only

Reporting checklist for protocol of a clinical trial.

Based on the SPIRIT guidelines.

Instructions to authors

Complete this checklist by entering the page numbers from your manuscript where readers will find each of the items listed below.

Your article may not currently address all the items on the checklist. Please modify your text to include the missing information. If you are certain that an item does not apply, please write "n/a" and provide a short explanation.

Upload your completed checklist as an extra file when you submit to a journal.

In your methods section, say that you used the SPIRITreporting guidelines, and cite them as:

Chan A-W, Tetzlaff JM, Gøtzsche PC, Altman DG, Mann H, Berlin J, Dickersin K, Hróbjartsson A, Schulz KF, Parulekar WR, Krleža-Jerić K, Laupacis A, Moher D. SPIRIT 2013 Explanation and Elaboration: Guidance for protocols of clinical trials. BMJ. 2013;346:e7586

		Reporting Item	Page Number
**Administrative information**			
Title	#1	Descriptive title identifying the study design, population, interventions, and, if applicable, trial acronym	1

For peer review only

Trial registration	#2a	Trial identifier and registry name. If not yet registered, name of intended registry	3 and 14
Trial registration: data set	#2b	All items from the World Health Organization Trial Registration Data Set	3 and 14
Protocol version	#3	Date and version identifier	3
Funding	#4	Sources and types of financial, material, and other support	14
Roles and responsibilities: contributorship	#5a	Names, affiliations, and roles of protocol contributors	2
Roles and responsibilities: sponsor contact information	#5b	Name and contact information for the trial sponsor	14
Roles and responsibilities: sponsor and funder	#5c	Role of study sponsor and funders, if any, in study design; collection, management, analysis, and interpretation of data; writing of the report; and the decision to submit the report for publication, including whether they will have ultimate authority over any of these activities	14
Roles and responsibilities: committees	#5d	Composition, roles, and responsibilities of the coordinating centre, steering committee, endpoint adjudication committee, data management team, and	7-14

For peer review only

		other individuals or groups overseeing the trial, if applicable (see Item 21a for data monitoring committee)	
**Introduction**			
Background and rationale	#6a	Description of research question and justification for undertaking the trial, including summary of relevant studies (published and unpublished) examining benefits and harms for each intervention	5-6
Background and rationale: choice of comparators	#6b	Explanation for choice of comparators	5-6
Objectives	#7	Specific objectives or hypotheses	6
Trial design	#8	Description of trial design including type of trial (eg, parallel group, crossover, factorial, single group), allocation ratio, and framework (eg, superiority, equivalence, non-inferiority, exploratory)	5-6
**Methods: Participants, interventions, and outcomes**			
Study setting	#9	Description of study settings (eg, community clinic, academic hospital) and list of countries where data will be collected. Reference to where list of study sites can be obtained	7

For peer review only

Eligibility criteria	#10	Inclusion and exclusion criteria for participants. If applicable, eligibility criteria for study centres and individuals who will perform the interventions (eg, surgeons, psychotherapists)	8
Interventions: description	#11a	Interventions for each group with sufficient detail to allow replication, including how and when they will be administered	8-13
Interventions: modifications	#11b	Criteria for discontinuing or modifying allocated interventions for a given trial participant (eg, drug dose change in response to harms, participant request, or improving / worsening disease)	8-12
Interventions: adherance	#11c	Strategies to improve adherence to intervention protocols, and any procedures for monitoring adherence (eg, drug tablet return; laboratory tests)	8-14
Interventions: concomitant care	#11d	Relevant concomitant care and interventions that are permitted or prohibited during the trial	NA
Outcomes	#12	Primary, secondary, and other outcomes, including the specific measurement variable (eg, systolic blood pressure), analysis metric (eg, change from baseline, final value, time to event), method of aggregation (eg, median, proportion), and time point for each outcome. Explanation of the clinical relevance of chosen efficacy and harm outcomes is strongly recommended	11-12

For peer review only

Participant timeline	#13	Time schedule of enrolment, interventions (including any run-ins and washouts), assessments, and visits for participants. A schematic diagram is highly recommended (see Figure)	8-11
Sample size	#14	Estimated number of participants needed to achieve study objectives and how it was determined, including clinical and statistical assumptions supporting any sample size calculations	12
Recruitment	#15	Strategies for achieving adequate participant enrolment to reach target sample size	9-12
**Methods: Assignment of interventions (for controlled trials)**			
Allocation: sequence generation	#16a	Method of generating the allocation sequence (eg, computer-generated random numbers), and list of any factors for stratification. To reduce predictability of a random sequence, details of any planned restriction (eg, blocking) should be provided in a separate document that is unavailable to those who enrol participants or assign interventions	NA
Allocation concealment mechanism	#16b	Mechanism of implementing the allocation sequence (eg, central telephone; sequentially numbered, opaque,	NA

For peer review only

		sealed envelopes), describing any steps to conceal the sequence until interventions are assigned	
Allocation: implementation	#16c	Who will generate the allocation sequence, who will enrol participants, and who will assign participants to interventions	NA
Blinding (masking)	#17a	Who will be blinded after assignment to interventions (eg, trial participants, care providers, outcome assessors, data analysts), and how	NA
Blinding (masking): emergency unblinding	#17b	If blinded, circumstances under which unblinding is permissible, and procedure for revealing a participant's allocated intervention during the trial	NA
**Methods: Data collection, management, and analysis**			
Data collection plan	#18a	Plans for assessment and collection of outcome, baseline, and other trial data, including any related processes to promote data quality (eg, duplicate measurements, training of assessors) and a description of study instruments (eg, questionnaires, laboratory tests) along with their reliability and validity, if known. Reference to where data collection forms can be found, if not in the protocol	11-13

For peer review only

Data collection plan: retention	#18b	Plans to promote participant retention and complete follow-up, including list of any outcome data to be collected for participants who discontinue or deviate from intervention protocols	NA
Data management	#19	Plans for data entry, coding, security, and storage, including any related processes to promote data quality (eg, double data entry; range checks for data values). Reference to where details of data management procedures can be found, if not in the protocol	13
Statistics: outcomes	#20a	Statistical methods for analysing primary and secondary outcomes. Reference to where other details of the statistical analysis plan can be found, if not in the protocol	12-13
Statistics: additional analyses	#20b	Methods for any additional analyses (eg, subgroup and adjusted analyses)	13
Statistics: analysis population and missing data	#20c	Definition of analysis population relating to protocol non-adherence (eg, as randomised analysis), and any statistical methods to handle missing data (eg, multiple imputation)	NA

Methods: Monitoring

Data monitoring: formal committee	#21a	Composition of data monitoring committee (DMC); summary of its role and reporting structure; statement of whether it is independent from the sponsor and competing interests; and reference to where further	13

For peer review only

		details about its charter can be found, if not in the protocol. Alternatively, an explanation of why a DMC is not needed	
Data monitoring: interim analysis	#21b	Description of any interim analyses and stopping guidelines, including who will have access to these interim results and make the final decision to terminate the trial	NA
Harms	#22	Plans for collecting, assessing, reporting, and managing solicited and spontaneously reported adverse events and other unintended effects of trial interventions or trial conduct	13-14
Auditing	#23	Frequency and procedures for auditing trial conduct, if any, and whether the process will be independent from investigators and the sponsor	NA
**Ethics and dissemination**			
Research ethics approval	#24	Plans for seeking research ethics committee / institutional review board (REC / IRB) approval	13-14
Protocol amendments	#25	Plans for communicating important protocol modifications (eg, changes to eligibility criteria, outcomes, analyses) to relevant parties (eg, investigators, REC / IRBs, trial participants, trial registries, journals, regulators)	NA

For peer review only

Consent or assent	#26a	Who will obtain informed consent or assent from potential trial participants or authorised surrogates, and how (see Item 32)	13-14
Consent or assent: ancillary studies	#26b	Additional consent provisions for collection and use of participant data and biological specimens in ancillary studies, if applicable	NA
Confidentiality	#27	How personal information about potential and enrolled participants will be collected, shared, and maintained in order to protect confidentiality before, during, and after the trial	13-14
Declaration of interests	#28	Financial and other competing interests for principal investigators for the overall trial and each study site	14
Data access	#29	Statement of who will have access to the final trial dataset, and disclosure of contractual agreements that limit such access for investigators	13
Ancillary and post trial care	#30	Provisions, if any, for ancillary and post-trial care, and for compensation to those who suffer harm from trial participation	NA
Dissemination policy: trial results	#31a	Plans for investigators and sponsor to communicate trial results to participants, healthcare professionals, the public, and other relevant groups (eg, via publication, reporting in results databases, or other data sharing arrangements), including any publication restrictions	13-14

For peer review only

Dissemination policy: authorship	#31b	Authorship eligibility guidelines and any intended use of professional writers	NA
Dissemination policy: reproducible research	#31c	Plans, if any, for granting public access to the full protocol, participant-level dataset, and statistical code	14

Appendices

Informed consent materials	#32	Model consent form and other related documentation given to participants and authorised surrogates	NA
Biological specimens	#33	Plans for collection, laboratory evaluation, and storage of biological specimens for genetic or molecular analysis in the current trial and for future use in ancillary studies, if applicable	NA

The SPIRIT Explanation and Elaboration paper is distributed under the terms of the Creative Commons Attribution License CC-BY-NC. This checklist was completed on 18. December 2023 using https://www.goodreports.org/, a tool made by the EQUATOR Network in collaboration with Penelope.ai

For peer review only

BMJ Open

Shared decision making for nonoperative management versus operative management of hip fractures in selected frail older adults with a limited life expectancy; a protocol for a nationwide implementation study

Journal:	*BMJ Open*
Manuscript ID	bmjopen-2023-083429.R1
Article Type:	Protocol
Date Submitted by the Author:	28-Feb-2024
Complete List of Authors:	Zeelenberg, Miliaan; Erasmus MC, Trauma research unit, Dept Surgery<br>Oosterwijk, Paulieke; Sint Antonius Ziekenhuis, Department of Trauma Surgery<br>Willems, Hanna; University of Amsterdam, Department of Geriatrics<br>Gosens, Taco; Elisabeth-TweeSteden Ziekenhuis, Department of Orthopaedic Surgery<br>Den Hartog, D; Erasmus MC, Department of Trauma Surgery<br>Joosse, Pieter ; Noordwest Ziekenhuisgroep, Department of Trauma Surgery<br>Loggers, Sverre; Noordwest Ziekenhuisgroep, Department of Trauma Surgery<br>Nijdam, Thomas ; Sint Antonius Ziekenhuis, Department of Surgery<br>Pel-Littel, Ruth; Vilans<br>Polinder, Suzanne; Erasmus MC, Department of Public Health<br>Jan Schuijt, Henk ; Sint Antonius Hospital, Department of Trauma Surgery<br>Wijnen, Hugo; Rijnstate, Department of Clinical geriatrics<br>Van der Velde, Detlef; Sint Antonius Ziekenhuis, Department of Surgery<br>Van Lieshout, Esther M.M.; Erasmus MC, Trauma Research Unit dept of Surgery<br>Verhofstad, Michael; Erasmus MC, Trauma Surgery<br>, PNOM-Implementation Study Group; PNOM-Implementation study group
**Primary Subject Heading**:	Surgery
Secondary Subject Heading:	Geriatric medicine, Palliative care, Patient-centred medicine
Keywords:	Frailty, Hip < ORTHOPAEDIC & TRAUMA SURGERY, Implementation Science, ORTHOPAEDIC & TRAUMA SURGERY, GERIATRIC MEDICINE

SCHOLARONE™ Manuscripts

Shared decision making for nonoperative management versus operative management of hip fractures in selected frail older adults with a limited life expectancy; a protocol for a nationwide implementation study

Miliaan L. Zeelenberg MD[1,*], Paulieke C. Oosterwijk MD[2,*], Hanna C. Willems MD PhD[3], Taco Gosens MD PhD[4], Dennis Den Hartog MD PhD[1], Pieter Joosse MD PhD[5], Sverre A.I. Loggers MD[1,5], Thomas M.P. Nijdam MD[2], Ruth E. Pel-Littel MD PhD[6], Suzanne Polinder PhD[7], Henk Jan Schuijt MD PhD[2], Hugo H. Wijnen MD[8], Detlef Van der Velde MD PhD[2], Esther M.M. Van Lieshout PhD MSc[1], Michael H.J. Verhofstad MD PhD[1], NOM-Implementation study group#

[1]Trauma Research Unit Department of Surgery, Erasmus MC, University Medical Center Rotterdam, Rotterdam, The Netherlands

[2]Department of Trauma surgery, St. Antonius Hospital, Utrecht, The Netherlands

[3]Department of Internal Medicine and Geriatrics, Amsterdam University Medical Center, Amsterdam, The Netherlands

[4]Department of Orthopedics, Elisabeth Hospital (ETZ), Tilburg, The Netherlands

[5]Department of Surgery, Noordwest Hospital group, Alkmaar, The Netherlands

[6]Vilans, Center of Expertise for Long-Term Care, Utrecht, The Netherlands.

[7]Department of Public Health, Erasmus MC, University Medical Center Rotterdam, Rotterdam, the Netherlands

[8]Department of Clinical Geriatrics, Rijnstate Hospital, Arnhem, The Netherlands

*Both authors contributed equally

For peer review only - http://bmjopen.bmj.com/site/about/guidelines.xhtml

NOM-Implementation study group is listed in Online supplementary materials 1.

Corresponding author

Dr. E.M.M. Van Lieshout

Trauma Research Unit Department of Surgery

Erasmus MC, University Medical Center Rotterdam

P.O. Box 2040

3000 CA Rotterdam

The Netherlands

Phone: +31.10.7031050

Mail: e.vanlieshout@erasmusmc.nl

Word count: 3541 words

For peer review only

Abstract

Background and purpose: Recent research has highlighted nonoperative management (NOM) as a viable alternative for frail older adults with hip fractures in the final phase of life. This study aims to guide Dutch physicians and hospitals nationwide in a standardized implementation of shared decision making (SDM) regarding surgery or NOM in selected frail older adults with a hip fracture.

Methods and analysis: The patient population for implementation includes frail older adults aged ≥70 years with an acute proximal femoral fracture, nursing home care or a similar level of care elsewhere, and at least one additional criterion (i.e. malnutrition, severe mobility impairment, or ASA ≥4). The two-year implementation study will be conducted in four phases. In phases 1 and 2, barriers and facilitators for implementation will be identified and an implementation protocol, educational materials, and patient information will be developed. Phase 3 will involve an implementation pilot in 14 hospitals across the Netherlands. The protocol and educational material will be improved based on healthcare provider and patient experiences gathered through interviews. Phase 4 will focus on upscaling to nationwide implementation and the effect of the implementation on NOM rate will be measured using data from the Dutch Hip Fracture Audit.

Ethics and dissemination: The study was exempted by the local Medical Research Ethics Committee (MEC-2023-0270, 10-05-2023) and Medical Ethics Committee United (W23.083, 26-04-2023). The study's results will be submitted to an open access international peer-reviewed journal. It's protocols, tools and results will be presented at several national and international academic conferences of relevant orthogeriatric (scientific) associations.

For peer review only

Trial registration number: NCT06079905 at ClinicalTrial.gov (11-10-2023)

For peer review only - http://bmjopen.bmj.com/site/about/guidelines.xhtml

Article summary

Strengths and limitations of this study

- This study builds on an existing foundation of recent well-received (inter)national publications, countrywide initiatives involving shared-decision making, and recent demands for assistance in the implementation of nonoperative management of hip fractures in selected frail older adults with a limited life-expectancy.
- The study will employ a staged approach in four phases, involving implementation strategies at the individual, local, and national level, that range from local educational sessions to nationwide distribution at conferences and proposed guideline changes.
- The study design allows for regular adjustment of its implementation protocols and educational materials based on patient and health care provider feedback from interviews from 14 different hospitals throughout the Netherlands.
- While the study has an ambitious 2-year time scheme to achieve nationwide implementation in the hospital setting, it will provide an important foundation for further initiatives.

For peer review only - http://bmjopen.bmj.com/site/about/guidelines.xhtml

Introduction

A hip fracture in frail older adults can indicate an approaching end of life. Advanced age, multiple comorbidities, and severe mobility limitations are important risk factors for early post-operative mortality after a hip fracture (1, 2). Studies show that >30% of patients die within one year post hip fracture, with six-month mortality rates in older adults with dementia exceeding 55%, often with low quality of life in the months preceding death (3-5). These patients, in their final phase of life, often do not benefit from an operative treatment that aims to restore mobility. Operations are often conducted in this population as a palliative treatment option, focused primarily on pain reduction. Based on these insights, a paradigm shift is occurring in clinical approach, emphasizing the importance of individualized care plans focused on optimal quality of life (6, 7).

The FRAIL-HIP study has confirmed that nonoperative management (NOM) can be a good alternative to operative management in select frail older adults with a limited life-expectancy (5, 8-10). Quality of life after NOM was valued non-inferiorly and the majority of the next of kin rated the quality of dying of their relative as very good (5, 8). Despite higher mortality rates in patients opting for NOM, the option to allow patients to pass away in a familiar environment was a compelling factor in favor of NOM in this select population (11). Both operative and nonoperative management can thus be effective treatment options as part of palliative care in selected frail older adults with a limited life-expectancy. Following these results, multiple Dutch hospitals have asked for guidance on implementing NOM in their institutions.

Central to NOM is the shared decision making (SDM) process, involving patients and their families in treatment decisions aligned with the patients' preferences and needs. Based on the

For peer review only - http://bmjopen.bmj.com/site/about/guidelines.xhtml

results of the FRAIL-HIP study and other literature, an implementation protocol has been drafted, piloted, and refined based on experiences of healthcare professionals (7, 9, 12). This protocol version will serve as the starting point for the iterative process towards nationwide implementation.

The aim of this study is nationwide implementation of SDM on treatment of hip fractures, involving both NOM and operative treatment, to ensure that frail older adults receive optimal, patient-centered care in the final phase of their lives.

For peer review only

Methods and analysis

Study design and aims

This two-year implementation study will consist of four phases (Figure 1); development of the implementation protocol and educational material (Phase 1 & 2), implementation across a subset of hospitals across the Netherlands and process evaluation and optimization of the protocol based on healthcare provider/patient experiences (Phase 3), and upscaling towards nationwide implementation and project evaluation (Phase 4). The study design of phases 3 and 4 entails a multicenter prospective cohort study. Overall, the project will cover all activities as recommended in the ImpRes Guide, which mentions four implementation phases (*i.e.*, exploration, installation, initial implementation, and full implementation) and should efficiently translate local knowledge into general knowledge (13). The 'Consolidated Framework for Implementation Research' (CFIR) was used for designing this project (14, 15). With education being a critical part of the implementation, elements of the education strategy from Wensing *et al.* (16) were added. The CFIR domains that the four phases of this project will cover are mentioned in Table 1.

To achieve the primary aim, this study will use a staged approach in four phases that involves three levels: The healthcare provider specific/local hospital level (micro) in phases 1-3, the level of a limited number of hospitals (meso) in phase 3, and the national level (macro) in phase 4. The four consecutive phases of the project have phase-specific aims with corresponding target populations and points of action, that are described in Table 2. To ensure nationwide implementation many parties and interest groups are or will be involved in the project (Table 3).

For peer review only - http://bmjopen.bmj.com/site/about/guidelines.xhtml

Study population

Phases 1 and 2 will not include patients or patient data but will include healthcare provider and patient association perspectives. Phases 3 and 4 will also include frail older adults with a limited life expectancy who sustained a proximal femoral fracture. Patients will be included and receive SDM on NOM and operation if they meet the following criteria:

1. Acute proximal femoral fracture (AO type 31), confirmed on X-ray or CT-scan
2. 70 years or older
3. Living in a nursing home or receiving similar care at home
4. And at least one of the following characteristics:
    - Malnutrition (cachexia or BMI <18.5 kg/m2)
    - Functional ambulation category* (FAC) 2 or lower
    - Severe comorbidities (ASA category 4 or 5)

Patients and/or proxies that do not have sufficient comprehension of the Dutch language will be excluded from the interviews during phase 3.

*The Functional Ambulation Categories (FAC) is a functional walking test that evaluates ambulation ability (17). This 6-point scale assesses ambulation status by determining how much human support the patient requires when walking, regardless of whether or not they use a personal assistive device. FAC ≤2 means that a patients at least has the need for (intermittent) help of another person to be able to ambulate (FAC 2) to no functional ambulatory capabilities (FAC 0).

Implementation in four phases

For peer review only - http://bmjopen.bmj.com/site/about/guidelines.xhtml

Phase 1 (exploration): evaluate current practice and identify facilitators and barriers of NOM implementation

Based on the evidence-based 'Measurement Instrument for Determinants of Innovations' (MIDI) (18, 19) and the 'Barriers and Facilitators Assessment Instrument' (BFAI) (20) a survey has been developed, aimed to identify the facilitators and barriers for applying SDM and NOM for selecting the best treatment for individual patients of the target population and identified five barriers and 23 facilitators (9). The patient perspective was not included in this study, but was explored in another study (11). A panel from older adults organizations, general practitioners and geriatric specialists will discuss and supplement the data from the aforementioned studies from their perspective.

Phase 2 (installation): developing an implementation strategy for NOM

A draft protocol for NOM implementation, with as focal point the SDM interview, has been written based on the experience and expertise of the project members, previous studies, and questionnaires. This protocol contains the central vision, the requirements for the implementation of NOM in selected frail older adults, and a recommendation for the composition of a local project team of stakeholders and drivers of change. To remove barriers, practical tools such as pocket cards, posters, factsheets, and local training options will be drafted or updated by the project team, based on the 'Expert Recommendations for Implementing Change strategies' (21). This should help drivers of change in achieving implementation goals and change day-to-day policies. The NOM implementation protocol will contain an overview of the data to be collected for NOM evaluation. A reporting tool will be developed in the Dutch Hip Fracture Audit (DHFA) for this purpose. In addition, educational modules for healthcare professionals will be

For peer review only - http://bmjopen.bmj.com/site/about/guidelines.xhtml

developed using the NOM protocol in combination with existing educational modules on SDM (22-24). NOM as a treatment option for selected frail older adults will also be included in the obligatory curriculum for residents by the Association of Surgeons of the Netherlands (in Dutch: Nederlandse Vereniging voor Heelkunde, NVvH), in order to incorporate NOM into the educational program of (orthopedic) trauma residents.

PHASE 3 (initial implementation): NOM-implementation in selected hospitals

NOM for selected frail older adults will be implemented in 14 selected hospitals (Figure 2, collaborators listed in Online supplementary materials 1). Based on the experience of these hospitals, the NOM implementation protocol and/or the educational material will be optimized. To safeguard implementation of NOM in the long term, the NOM protocol pays explicit attention to the involvement of all relevant healthcare providers by informing those involved before, during and after implementation, and by collecting data on relevant feasibility parameters as described in the Bowen model (25). To achieve this, the site principal investigator will be asked to establish a local project team. The team should represent all groups involved in NOM, including relevant physicians and, as applicable, residents of the involved departments, specialized nurses, physician assistants, and hospital management. Prior to implementation, this local team will meet with the researchers for an interview to train them in applying the SDM and the NOM implementation protocol, and to collect details on their specific needs for the implementation.

The local team will perform patient selection and apply SDM on NOM and operative treatment for all patients who meet the eligibility criteria. After the start of the implementation, during the first six months, patients, relatives, and/or next of kin will be approached once by

For peer review only - http://bmjopen.bmj.com/site/about/guidelines.xhtml

telephone to ask about their satisfaction with the treatment decision process, and the chosen treatment. This will be done through a semi-structured interview. To provide more insight about their own patient population the interview results will be discussed with the participating centers on a quarterly basis. All collected data will be used to improve the NOM protocol and/or educational material prior to phase 4. Six months after start of implementation, the local team will be approached again for an interview to evaluate the process with the relevant parameters: adoption, integration, applicability, expansion, and early local effectiveness.

PHASE 4 (nationwide implementation): NOM-implementation in all hospitals in the Netherlands and project analysis

Phase 4 is an expansion of phase 3 to reach nationwide implementation. The implementation strategy will essentially be the same as in phase 3. Due to the increase in number of hospitals, the supervision and contact with the researchers and steering committee will be less intensive than in phase 3. In practice, this means that the NOM protocol will be implemented, but the interviews with health care professionals in the hospitals (prior to start and six months after start) will be omitted. To ensure that all information on SDM and NOM implementation is available continuously to both patients and healthcare providers, a dedicated website will be developed and maintained. To effectively reach a broad audience of healthcare providers and keep them updated on the implementation process and learning experiences, our goal is to give presentations at the annual meetings of orthogeriatric scientific associations, as well as associations for general practitioners and elderly care physicians (Table 3). In addition, time slots for presentations will be requested at the meetings of the regional trauma networks. Ultimately, we aim to include SDM on NOM or operative treatment for proximal femoral fractures in select

For peer review only - http://bmjopen.bmj.com/site/about/guidelines.xhtml

frail older adults in the National guidelines for hip fracture treatment and operative care in older adults.

Outcome measures and data collection

The primary outcome measure (for phase 3 and 4) is (change in) the NOM rate. Type of treatment (NOM versus operative treatment) will be extracted from the Dutch Hip Fracture Audit (DHFA). The DHFA is a national database, including over 95% of Hip fracture patients, and can provide automated anonymous patient data up to one year after hip fracture treatment. To evaluate the effect of the implementation, the change in NOM rate and operative rate for the targeted population will be evaluated and compared with the rate prior to implementation. This will be calculated using the annual national DHFA data from the two-years preceding implementation per hospital, as baseline. The post-implementation NOM rate will be collected immediately after completion of phase 4, as well as at the end of 2025. The secondary outcome measures (for phase 3 only) will be collected during semi-structured interviews with patients/proxy or next of kin, four to six weeks after inclusion. These are the satisfaction with the chosen treatment on a numeric rating scale (0-10), their valuation of the information received during the SDM process, and if the patient's needs were respected enough in the treatment decision. The interview sheets (in Dutch), used for patient or healthcare provider interviews in phases 1-3, are included in online supplementary materials 2.

The following patient data will be also collected in phases 3 and 4 from the DHFA at 3-months and one year after hip fracture treatment (emerging from the patients' medical records). Patient characteristics (age, sex, ASA, Functional Ambulation Category), pre-fracture situation (residence, mobility, KATZ Activities of Daily Living (ADL) score), fracture details (fracture

For peer review only - http://bmjopen.bmj.com/site/about/guidelines.xhtml

classification, affected side), hospital details (hospital length of stay and intensive care length of stay), and outcome details (complications, revision surgeries, readmission, 30-day mortality, and time until death).

Data will be collected for each patient who meets the eligibility criteria. To reduce the administrative burden, the DHFA database will be used as much as possible. Data from the DHFA will be imported in a Castor EDC database, in which hospitals can only see their own data. The datasets of all hospitals will be combined for the final analysis.

Sample size calculation

A formal sample size calculation was not made as the primary aim is implementation on a national level. To give an idea of the total national population size the following estimate was made: In 2020 21,165 patients presented to a hospital in the Netherlands with a proximal femoral fracture (26). Of these 17,225 (81.5%) were aged 70 years or older. An approximate 25% of this population lived in a nursing home or other institution. Which projects to about 4,300 patients as the primary target population of this study. Approximately half of these patients will meet our inclusion criteria and thus not all patients will be eligible for NOM, but this gives a broad estimate of the annual number of patients that should at least be considered for SDM on NOM and operative treatment.

Statistical analysis

Data will be analyzed using the Statistical Package for the Social Sciences (SPSS) version 28 or higher (SPSS, Chicago, Ill., USA). Normality of continuous data will be tested with the Shapiro-

For peer review only - http://bmjopen.bmj.com/site/about/guidelines.xhtml

Wilk test, and homogeneity of variances will be tested using the Levene's test. A p-value <0.05 will be taken as threshold of statistical significance. Missing values will not be imputed.

The primary outcome, NOM rate, will be determined by dividing the number of patients treated with NOM by the total eligible number of patients (per participating center and for all participating centers combined).

All continuous data will be reported as mean with standard deviation (SD; if normally distributed based on Shapiro-Wilk test) or as median with quartiles (if not normally distributed). Categorical data will be reported as number with percentage. Pre- and post-implementation data will be compared with a Student T-test (continuous data, normally distributed), Mann-Whitney U-test (continuous data, non-normally distributed), or a Chi-square or Fisher's Exact test (categorical data), as applicable. The analysis will be stratified by NOM versus operative treatment, and further stratified as feasible depending on the final study population, e.g., by time since NOM implementation, pre-fracture mobility, ADL status, fracture classification, or bilateral fracture.

Project timeline

Phase 1 of the study started in June 2023 and ended in August 2023. Phase 2 started in June 2023 and ended in October 2023. Phase 3 started in September 2023 and will approximately end in October 2024. Phase 4 will start in June 2024 and will approximately be concluded in June 2025.

Patient and public involvement

A patient association representative was consulted during the drafting of the study protocol and could provide input for the study design and implementation tools. Through interviews, study

For peer review only - http://bmjopen.bmj.com/site/about/guidelines.xhtml

participants (patients, next-of-kin and healthcare providers) will be directly involved in providing feedback on the SDM process and educational tools used during the project. In addition to general publication, a summary of the main results will be made available to study participants on request.

For peer review only

For peer review only - http://bmjopen.bmj.com/site/about/guidelines.xhtml

Ethics and dissemination

Ethical approval, risks and dissemination

The study was exempted by the local Medical Research Ethics Committee (MEC-2023-0270, 10-05-2023) and Medical Ethics Committee United (W23.083, 26-04-2023) and is registered at ClinicalTrial.gov (NCT06079905, 11-10-2023). The study was approved by the board of directors of all hospitals participating in phase 3. There are no risks involved in this study. The only burden is the time needed for the interviews with health care providers and patients/next of kin in phase 3. The interviews will be performed by researchers following an opt-out design, participants can stop at any time. Participants will be asked to consent to share their contact details with the researchers and participation means interview data can be used (Online supplementary materials 3). Besides this, only already existing and routinely collected data will be collected. All treatments, chosen after SDM, will be performed following local standard care protocols. Based on the above, the overall burden and risks are considered negligible. The study will follow Good Clinical Practice (GCP) guidelines. The study's results will be submitted to an open access international peer-reviewed journal. As previously explained above, it's protocols, tools and results will be made publicly available and presented at several national and international academic conferences of relevant orthogeriatric (scientific) associations.

Data management, monitoring, and privacy

All data and information collected regarding this study will be treated confidentially by the researchers. Research data that can be traced to individual persons can only be viewed by authorized personnel (members of the research team and members of the health care inspection).

For peer review only - http://bmjopen.bmj.com/site/about/guidelines.xhtml

Data will be encoded and stored in a Castor EDC database with password restricted access to the researchers at individual sites only. Regarding participants privacy, the Dutch 'General data protection regulation (GDPR) implementation act' will be applied.

Funding and disclosures

This project has been funded by the Netherlands Organization for Health Research and Development (ZonMw; project No. 10390162210008). The funder of the study has no role in study design, data collection, data analysis, data interpretation, or writing of the manuscript. All authors declare no conflicts of interest or competing interests for this project.

For peer review only - http://bmjopen.bmj.com/site/about/guidelines.xhtml

Discussion

Clinical impact

This project provides an important translation of recent scientific publications to clinical practice and its success could play an important role in (inter)national surgical and geriatric guideline changes. Successful implementation of NOM will change the way healthcare providers view acute hip fractures in frail older adults with a limited life-expectancy. Instead of primarily focusing on clinical possibility of hip fracture treatment, the focus will shift towards treatment concerning patients' quality of life, harm reduction, and effective palliative care in the final stages of life. Because of this shift, the NOM rate is expected to rise in this select group. However, the main goal is not to increase NOM rate, but to provide each patient with the clinical care that best suits their wishes, needs, and clinical reality. Nationwide implementation of SDM on NOM will also lead to a reduction of overtreatment and might reduce associated costs (10). Another important outcome is reduced practice variation between hospitals in both patient selection for (SDM on) NOM and nationwide implementation of a universal structured SDM protocol for frail older adults with a hip fracture.

Strengths and limitations

The strength of this study is that it builds on an existing foundation of recent well-received (inter)national publications, countrywide initiatives involving SDM and advance care planning, and recent demands for assistance in the implementation of NOM from several hospitals. The project group includes experienced professionals from (orthopedic-) trauma surgery, geriatrics, epidemiology, and implementation science from both university medical centers and general

For peer review only - http://bmjopen.bmj.com/site/about/guidelines.xhtml

hospitals, supplemented with patient perspectives and experts in SDM and study methodology. A limitation of the project is the limited time period for the initial project. Achieving nationwide implementation within two years, which is the maximum allowed project duration of the grantor, might be an overoptimistic aim. However, the project will have developed a strong foundation on which future projects can build and will keep sharing its tools and information, even after completion. Another limitation is that the inclusion criteria might prove to be too narrow. There might be a larger group of patients for whom NOM could suit their preferences or goals of care. However, the project group has decided to keep strict criteria to prevent the pendulum swinging too far and causing undertreatment with long-term survival without functional recovery after opting for nonoperative treatment, which results in potential decreased quality of life and associated adverse events. Depending on the results of the initial implementation the inclusion criteria in the protocol might be adjusted to suit the needs of patients and health care providers. Finally, the NOM rate in the DHFA database might be an underestimation, as patients living in a nursing home might already have advance care plans preventing them from being transferred to the hospital in the case of a hip fracture. As the NOM implementation gains national traction more attention to advance care planning around hip fractures might lead to an unmeasured increase of the NOM rate in the pre-hospital setting. While reducing DHFA registration for this project, improved communication on patients wishes and integration of hip fractures in advance care planning, between patients and (geriatric) medical practitioners within and outside the hospital, is a desired effect that will improve the quality of NOM and patients' treatment satisfaction.

Future perspective

For peer review only - http://bmjopen.bmj.com/site/about/guidelines.xhtml

The success of this project can be used as an international example for trauma care in selected frail older adults and lead to further changes in advance care planning within this older trauma population. The projects finished protocols and clinical tools may be used for updating the Dutch national 'Guideline for the treatment of proximal femoral fractures' and 'Guideline for multidisciplinary treatment of operative treatment of frail older adults' and may also help updating international treatment guidelines on proximal femoral fractures in selected frail older adults with a limited life-expectancy.

For peer review only

Statements

Funding

This project was previously peer-reviewed and accepted for funding by the Netherlands Organization for Health Research and Development (ZonMw; project No. 10390162210008)

Competing interest

All authors declare no conflicts of interest in relation to the current study. The funder of the study has no role in study design, data collection, data analysis, data interpretation, or writing of the manuscript.

Author contributions

MLZ, PCO, HCW, TG, DDH, PJ, SAIL, TMPN, REP, SP, HJS, HHW, DVDV, EMMVL, and MHJV were all involved in conceptualization and/or designing of the study, and the writing and revising of associated documents and protocols. MHJV will act as principal investigator. MLZ and PCO wrote the draft of the main manuscript. EMMVL and HJS provided direct supervision. MLZ, PCO, HCW, TG, DDH, PJ, SAIL, TMPN, REP, SP, HJS, HHW, DVDV, EMMVL, and MHJV actively contributed to critical revision of the manuscript in multiple revision rounds. The NOM-Implementation study group contains the representatives from each of the 14 participating hospitals. All authors have read and approved the final version of this manuscript.

For peer review only - http://bmjopen.bmj.com/site/about/guidelines.xhtml

For peer review only

References

1. Nijmeijer WS, Folbert EC, Vermeer M, Slaets JP, Hegeman JH. Prediction of early mortality following hip fracture surgery in frail elderly: The Almelo Hip Fracture Score (AHFS). Injury. 2016;47(10):2138-43.
2. Van de Ree CL, Gosens T, Van der Veen AH, Oosterbos CJ, Heymans MW, De Jongh MA. Development and validation of the Brabant Hip Fracture Score for 30-day and 1-year mortality. Hip Int. 2020;30(3):354-62.
3. Hu F, Jiang C, Shen J, Tang P, Wang Y. Preoperative predictors for mortality following hip fracture surgery: a systematic review and meta-analysis. Injury. 2012;43(6):676-85.
4. Morrison RS, Siu AL. Survival in end-stage dementia following acute illness. JAMA. 2000;284(1):47-52.
5. Loggers SAI, Van Balen R, Willems HC, Gosens T, Polinder S, Ponsen KJ, et al. The Quality of Dying in Frail Institutionalized Older Patients After Nonoperative and Operative Management of a Proximal Femoral Fracture: An In-Depth Analysis. Am J Hosp Palliat Care. 2023:10499091231180556.
6. Schuijt HJ, Lehmann LS, Javedan H, von Keudell AG, Weaver MJ. A Culture Change in Geriatric Traumatology: Holistic and Patient-Tailored Care for Frail Patients with Fractures. J Bone Joint Surg Am. 2021;103(18):e72.
7. Nijdam TMP, Laane D, Schiepers TEE, Smeeing DPJ, Kempen DHR, Willems HC, et al. The goals of care in acute setting for geriatric patients in case of a hip fracture. Eur J Trauma Emerg Surg. 2023;49(4):1835-44.
8. Loggers SAI, Willems HC, Van Balen R, Gosens T, Polinder S, Ponsen KJ, et al. Evaluation of Quality of Life After Nonoperative or Operative Management of Proximal Femoral Fractures in Frail Institutionalized Patients: The FRAIL-HIP Study. JAMA Surg. 2022;157(5):424-34.
9. Spronk I, Loggers SAI, Joosse P, Willems HC, Van Balen R, Gosens T, et al. Shared decision making for the treatment of proximal femoral fractures in frail institutionalized older patients: healthcare providers' perceived barriers and facilitators. Age Ageing (in print). 2022.

For peer review only - http://bmjopen.bmj.com/site/about/guidelines.xhtml

10. Loggers SAI, Geraerds A, Joosse P, Willems HC, Gosens T, Van Balen R, et al. Nonoperative versus operative management of frail institutionalized older patients with a proximal femoral fracture: a cost-utility analysis alongside a multicenter prospective cohort study. Osteoporos Int. 2023;34(3):515-25.

11. Nijdam TMP, Laane D, Spierings JF, Schuijt HJ, Smeeing DPJ, van der Velde D. Proxy-reported experiences of palliative, non-operative management of geriatric patients after a hip fracture: a qualitative study. BMJ Open. 2022;12(8):e063007.

12. Palliatief niet-operatief management bij kwetsbare oudere patiënten met een heupfractuur. Implementatiehandleiding Versie 1.1.1., d.d. 20-06-2022.

13. Center for Implementation Science Research. Implementation science research development (ImpRes) tool 2018 [Available from: https://impsci.tracs.unc.edu/wp-content/uploads/ImpRes-Guide.pdf. .

14. Damschroder LJ, Aron DC, Keith RE, Kirsh SR, Alexander JA, Lowery JC. Fostering implementation of health services research findings into practice: a consolidated framework for advancing implementation science. Implement Sci. 2009;4:50.

15. Birken SA, Powell BJ, Shea CM, Haines ER, Alexis Kirk M, Leeman J, et al. Criteria for selecting implementation science theories and frameworks: results from an international survey. Implement Sci. 2017;12(1):124.

16. Wensing M, Fluit C, Grimshaw J, Grol R. Educational Implementation Strategies. In Improving Patient Care (eds M. Wensing, R. Grol and J. Grimshaw. 2020.

17. Holden MK, Gill KM, Magliozzi MR, Nathan J, Piehl-Baker L. Clinical gait assessment in the neurologically impaired. Reliability and meaningfulness. Phys Ther. 1984;64(1):35-40.

18. Fleuren MA, Paulussen TG, Van Dommelen P, Van Buuren S. Towards a measurement instrument for determinants of innovations. Int J Qual Health Care. 2014;26(5):501-10.

19. Fleuren M, Paulussen T, Van Dommelen P, Van Buuren S. Measurement instrument for determinants of innovations (MIDI). Leiden: TNO. 2014.

20. Harmsen M, Peters M, Wensing M. Barriers and facilitators assessment instrument: Introduction, instructions and instrument. Nijmegen: IQ healthcare Scientific Institute for Quality of Healthcare. Radboud University Medical Center Nijmegen. 2005.

For peer review only - http://bmjopen.bmj.com/site/about/guidelines.xhtml

21. Powell BJ, Waltz TJ, Chinman MJ, Damschroder LJ, Smith JL, Matthieu MM, et al. A refined compilation of implementation strategies: results from the Expert Recommendations for Implementing Change (ERIC) project. Implement Sci. 2015;10:21.

22. Dialuge Trainer. Samen beslissen met ouderen met multimorbiditeit: Dialogue Trainer; [Available from: https://nl.dialoguetrainer.app/module/samenbeslissengeriatrie-open/.

23. FMS. Samen Beslissen (Shared decision making): Federatie Medisch Specialisten; 2023 [Available from: https://demedischspecialist.nl/themas/thema/samen-beslissen-0.

24. Kennisplatform Uitkomstgerichte Zorg. Samen beslissen: Kennisplatform Uitkomstgerichte Zorg; [Available from: https://www.platformuitkomstgerichtezorg.nl/themas/samen+beslissen/default.aspx.

25. Bowen DJ, Kreuter M, Spring B, Cofta-Woerpel L, Linnan L, Weiner D, et al. How we design feasibility studies. Am J Prev Med. 2009;36(5):452-7.

26. CBS. Hospital admissions and patient diagnosis (Ziekenhuisopnamen en -patiënten; diagnose-indeling): Centraal Bureau Statistiek (Dutch Central Bureau for Statistics) 2022 [updated 16-06-2022. Available from: https://opendata.cbs.nl/statline/#/CBS/nl/dataset/84069NED/table?ts=1670493690817.

For peer review only - http://bmjopen.bmj.com/site/about/guidelines.xhtml

Figures and Tables

Figure 1: Schematic overview of the implementation process in 4 phases

DHFA, Dutch Hip Fracture Audit; NOM, nonoperative management

Figure 2: Map of participating hospitals during the initial implementation in selected hospitals (Phase 3)

For peer review only

For peer review only - http://bmjopen.bmj.com/site/about/guidelines.xhtml

Table 1: CFIR domains and their role in the implementation project

CFIR Domain	Role in project
Inner setting	Gain insight into the hospital/implementation location, including internal network and communication, culture (norms and values), knowledge, current use of the intervention, and implementation climate (tension for change, feedback, learning climate)
Outer setting	Gain insight into the needs of patients/proxies and pressure from peers (need for hospitals to use the same protocol).
Characteristics of individuals	Gain insight into the confidence/preparedness to discuss and apply NOM through SDM with the intended target group
Characteristics of the intervention	Gain insight into the SDM conversation as part of NOM, the needs of healthcare providers on how to conduct SDM and who participates in it, and what they need to apply NOM.

CFIR, Consolidated Framework for Implementation Research; NOM, nonoperative management; SDM, shared decision making

For peer review only

Table 2: Study aims with specific action points per targeted group for the four study phases

Points of action per phase	Targeted group	Aim/outcome
Phase 1		
Qualitative research on patient experiences and perceived barriers and facilitators for SDM on NOM	Patients, next-of-kin and healthcare providers*	Enriching the implementation protocol with recent research on patient/healthcare provider experiences regarding SDM on NOM versus operative treatment.<br>Providing insight into barriers and facilitators on the local and individual level
Phase 2		
Development of educational material on SDM with frail older adults with hip fractures for patients and healthcare providers<br>Implementing SDM on NOM in the curriculum for (orthopedic) surgeons in training	Patients, next-of-kin and healthcare providers*	Increasing (practical) knowledge on performing SDM on NOM in healthcare providers.<br>Providing healthcare providers with tools to prepare patients and next-of-kin for SDM on NOM versus operative treatment
Phase 3		
Presentations of the Implementation protocol and included tools at annual meetings of the involved scientific associations	Healthcare providers*, members of associated scientific associations	Creating awareness and a sense of urgency for SDM implementation and identifying local drivers of change for future implementation
Implementation of the NOM implementation protocol and evaluation through interviews with patients and healthcare providers in 14 selected Dutch hospitals	Local project teams and drivers of change in 14 Dutch hospitals and patients/next-of-kin	Evaluating and improving the implementation protocol and educational modules based on local experiences, barriers and facilitators through patient- and healthcare provider interviews
Phase 4		
Maintaining and improving awareness through conference- and regional trauma network presentations<br>Identification of local ambassadors/drivers of change<br>Dissemination of the implementation protocol/educational tools through a dedicated website, scientific publications and media coverage	Health care providers*, and specifically, local drivers of change, in all Dutch hospitals treating frail older adults with hip fractures	Upscaling of the implementation to all hospitals in the Netherlands that treat the targeted patient group
Measuring the effect of implementation, changes in NOM rate, through the DHFA database	Dutch patients and hospitals through the DHFA database	Evaluating changes in practice after implementation

For peer review only - http://bmjopen.bmj.com/site/about/guidelines.xhtml

*Healthcare providers include (orthopedic) trauma surgeons, geriatricians, acute care physicians, anesthesiologists, residents, and physician assistants directly involved in (acute) care for frail older adults with a hip fracture in Dutch hospitals.

DHFA, Dutch Hip Fracture Audit; NOM, nonoperative management; SDM, shared decision making

For peer review only

For peer review only - http://bmjopen.bmj.com/site/about/guidelines.xhtml

Table 3: Participating parties and their roles and responsibilities

Participator(s)	Roles and responsibilities
Central project group/steering committee	The project group is responsible for the general oversight of the entire project.
Participating hospitals	They will implement the NOM protocol locally, and will provide data needed to evaluate the effect of the implementation. They will need to allow local training, and participate in interviews to gain insight into their experience with NOM implementation.
Patients and/or proxies	Will be asked to voluntarily participate in interviews and provide feedback on the chosen treatment and SDM process.
Dutch Hip Fracture Audit (DHFA) and Dutch Institute for Clinical Auditing (DICA)	They will provide the data so the hospitals can use it as mirror information. The DHFA data (which are existing data) will be used as pre and post implementation data, and will thus allow accurate monitoring of the impact of NOM implementation.
Scientific associations (Dutch Trauma Society (NVT), Dutch Association of Surgeons of the Netherlands (NVvH), Dutch Orthopedic Association (NOV), and Dutch Association of Clinical Geriatrics (NVKG))	The scientific associations are responsible for education for medical specialists and residents-in-training. They will play a key role in the education sessions, and in forwarding information on our project to their members.
Members of the National Association of General Practitioners (LHV) and the Association for Older Adult Care Physicians (Verenso)	Will be kept informed about this project through mailings and presentations during their annual conferences.

DHFA, Dutch Hip Fracture Audit; DICA, Dutch Institute Clinical Auditing; LHV, Landelijke Huisartsen Vereniging; NVKG, Nederlandse Vereniging voor Klinische Geriatrie; NVT, Nederlandse Vereniging voor Traumachirurgie; NVvH, Nederlandse Vereniging voor Heelkunde; NOV, Nederlandse Vereniging voor Orthopedie; NOM, nonoperative management; SDM, shared decision making;

For peer review only - http://bmjopen.bmj.com/site/about/guidelines.xhtml

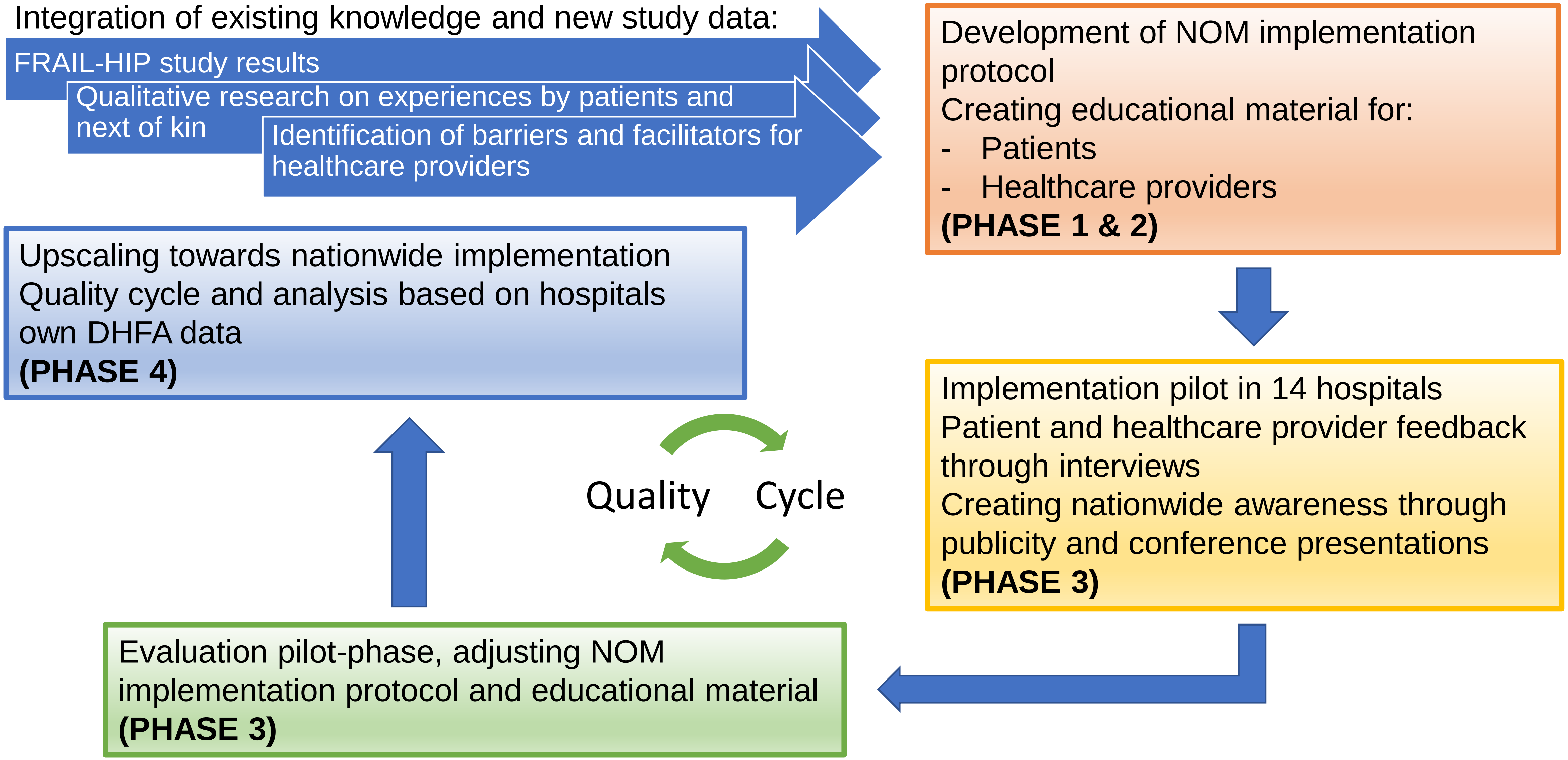
Integration of existing knowledge and new study data:
FRAIL-HIP study results
Qualitative research on experiences by patients and next of kin
Identification of barriers and facilitators for healthcare providers
Development of NOM implementation protocol
Creating educational material for:
- Patients
- Healthcare providers
(PHASE 1 & 2)
Implementation pilot in 14 hospitals
Patient and healthcare provider feedback through interviews
Creating nationwide awareness through publicity and conference presentations
(PHASE 3)
Evaluation pilot-phase, adjusting NOM implementation protocol and educational material
(PHASE 3)
Upscaling towards nationwide implementation
Quality cycle and analysis based on hospitals own DHFA data
(PHASE 4)
Quality
Cycle

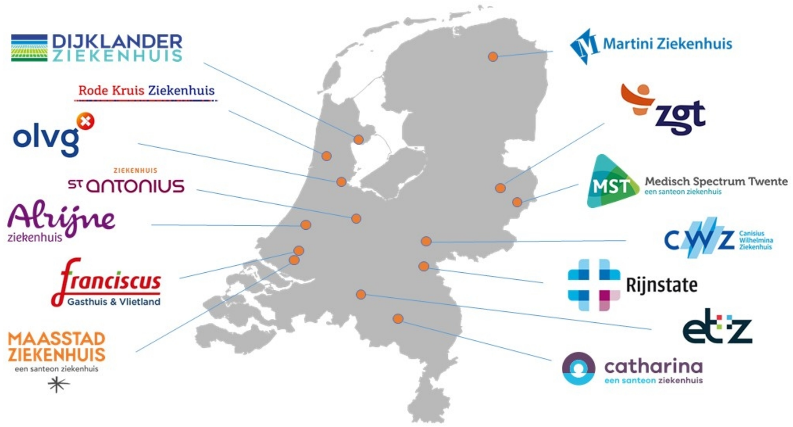

Figure 2: Map of participating hospitals during the initial implementation in selected hospitals (Phase 3)

338x190mm (96 x 96 DPI)

Online supplementary materials 1

Table S1: Contributors in NOM-Implementation study group

Institution	Location	First name	Last name	Title/Academic degree	Medical specialty
Alrijne ziekenhuis	Leiderdorp	E.	Sohl	MD PhD	Geriatrics
Alrijne ziekenhuis	Leiderdorp	J.A.	Jansen	MD	Orthopedics
Alrijne ziekenhuis	Leiderdorp	M.	Leijnen	MD PhD	Surgery
Carnisius Wilhelmina ziekenhuis	Nijmegen	W.A.H.	Van der Stappen	MD	Surgery
Carnisius Wilhelmina ziekenhuis	Nijmegen	J.	Jansen	MD PhD	Orthopedics
Carnisius Wilhelmina ziekenhuis	Nijmegen	M.	Peters-Kop	PA	Geriatric trauma unit
Catharina ziekenhuis	Eindhoven	A.H.	Van der Veen	MD PhD	Surgery
Catharina ziekenhuis	Eindhoven	N.C.	Schepel	MD	Orthopedics
Catharina ziekenhuis	Eindhoven	J.A.M.	Wilmer	MD	Geriatrics
Dijklander ziekenhuis	Hoorn	J.	Steens	MD PhD	Orthopedics
Dijklander ziekenhuis	Hoorn	J.	Winkelhagen	MD	Surgery
Dijklander ziekenhuis	Hoorn	N.M.F.	Noorda	MD	Geriatrics
Elisabeth TweeSteden ziekenhuis	Tilburg	H.A.A.M.	Maas	MD PhD	Geriatrics
Franciscus ziekenhuis	Rotterdam	O.	Wijers	MD PhD	Surgery
Franciscus ziekenhuis	Rotterdam	V.	Vis	PA	Geriatric trauma unit
Maasstad ziekenhuis	Rotterdam	D.	Van der Stap	MD	Geriatrics
Maasstad ziekenhuis	Rotterdam	G.R.	Roukema	MD PhD	Surgery
Martini ziekenhuis	Groningen	E.	Bosma	MD	Surgery
Martini ziekenhuis	Groningen	T.M.	Van Raaij	MD PhD	Orthopedics
Martini ziekenhuis	Groningen	A.M.	Van der Knaap	MD	Geriatrics
Medisch spectrum Twente	Enschede	R.	de Groot	MD PhD	Surgery
Medisch spectrum Twente	Enschede	A.V.C.M.	Zeegers	MD PhD	Orthopedics
OLVG	Amsterdam	H.A.	Formijne Jonkers	MD PhD	Surgery
OLVG	Amsterdam	D.H.R.	Kempen	MD PhD	Orthopedics
OLVG	Amsterdam	K.	De Vries	MD	Geriatrics
Rijnstate ziekenhuis	Arnhem	A.F.	Pull ter Gunne	MD PhD	Surgery
Rijnstate ziekenhuis	Arnhem	M.P.	Somford	MD PhD	Orthopedics
Rode Kruis ziekenhuis	Beverwijk	E.	Tanis	MD PhD	Surgery
Rode Kruis ziekenhuis	Beverwijk	J.H.	Duits	MD	Geriatrics
Rode Kruis ziekenhuis	Beverwijk	R.A.	Zandbergen	MD	Orthopedics
St. Antonius ziekenhuis	Nieuwegein	N.C.	Leegwater	MD PhD	Orthopedics

For peer review only

St. Antonius ziekenhuis	Nieuwegein	O.C.	Geraghty	MD PhD	Geriatrics
Ziekenhuisgroep Twente	Almelo	J.H.	Hegeman	MD PhD	Surgery
Ziekenhuisgroep Twente	Almelo	E.M.	Regtuijt	MD	Geriatrics

MD, Doctor of medicine; PA, Physician assistant; PhD, Doctor of philosophy

For peer review only

Datum (dd-mm-jjjj): ___ - ___ - 20___

Datasheet interviews phase 1

Introductie:

- Deelnemer(s) welkom heten en bedanken voor hun deelname.
- Doel van het interview toelichten.
- Benoemen dat deelname betekent dat de antwoorden gebruikt zullen worden om het PNOM-handleiding waar mogelijk te verbeteren. Dit zal te allen tijden anoniem zijn. Wie dit niet wil, de kans geven om het gesprek te beëindigen of de bijeenkomst te verlaten.

Om PNOM te implementeren zijn naast de FRAIL-HIP studie 2 zijstudies uitgevoerd.

1) Enquête van Spronk *et al*. in 2022 onder 271 zorgprofessionals in het ziekenhuis. Dit heeft geleid tot 5 belemmerende factoren en 23 bevorderende factoren voor PNOM implementatie.
2) Interviews van Nijdam *et al*. in 2022 onder nabestaanden van patiënten bij wie voor PNOM gekozen was.

Hebt u aanvullingen of suggesties na lezen van de conceptversie? Zo ja, zou u die hieronder willen vermelden?

A) Vertegenwoordigers huisartsen:

____

____

____

____

____

B) Vertegenwoordigers SOGs:

____

____

____

____

____

C) Vertegenwoordigers KBO-PCOB:

____

____

____

____

For peer review only - http://bmjopen.bmj.com/site/about/guidelines.xhtml

Erasmus MC Universitair Medisch Centrum Rotterdam — Amsterdam UMC Universitair Medische Centra — St Antonius een santeon ziekenhuis

Datum (dd-mm-jjjj): ___ - ___ - 20___

Datasheet interviews phase 2

Introductie:

- Deelnemer(s) welkom heten en bedanken voor hun deelname.
- Doel van het interview toelichten.
- Benoemen dat deelname betekent dat de antwoorden gebruikt zullen worden om de PNOM-handleiding waar mogelijk te verbeteren. Dit zal te allen tijden anoniem zijn. Wie dit niet wil, de kans geven om het gesprek te beëindigen of de bijeenkomst te verlaten.

Om PNOM te implementeren is een PNOM-handleiding gemaakt. Dit is gebaseerd op de resultaten van de FRAIL-HIP en nevenstudies naar beperkende en bevorderende factoren voor implementatie. Het concept protocol bevat o.a.:

1) Doel van de implementatie
2) Benodigd lokaal team
3) Procedure om implementatie te bewerkstelligen
4) Factsheet
5) Data die nodig zijn om het effect van de implementatie te evalueren

Hebt u aanvullingen of suggesties na lezen van de conceptversie? Zo ja, zou u die hieronder willen vermelden?

A) Input voor PNOM-handleiding:

___

B) Input voor patiëntinformatie:

___

C) Input voor zakkaart:

___

For peer review only - http://bmjopen.bmj.com/site/about/guidelines.xhtml

Erasmus MC Universitair Medisch Centrum Rotterdam
Amsterdam UMC Universitair Medische Centra
ST ANTONIUS een santeon ziekenhuis

Datum (dd-mm-jjjj): ___ - ___ - 20___

D) Input voor factsheet:

____
____
____
____
____

E) Overig input:

____
____
____
____
____

For peer review only

For peer review only - http://bmjopen.bmj.com/site/about/guidelines.xhtml

Datum (dd-mm-jjjj): ___ - ___ - 20___

Datasheet interviews phase 3 (nul-meting)

Introductie:
- Deelnemer(s) welkom heten en bedanken voor hun deelname.
- Doel van het interview toelichten.
- Benoemen dat deelname betekent dat de antwoorden gebruikt zullen worden om het PNOM protocol waar mogelijk te verbeteren. Dit zal te allen tijden anoniem zijn. Wie dit niet wil, de kans geven om het gesprek te beëindigen of de bijeenkomst te verlaten.

Uw ziekenhuis gaat deelnemen/neemt deel aan implementatie van PNOM voor een specifieke groep kwetsbare ouderen met een heupfractuur. Het protocol bevat o.a.:
1) Doel van de implementatie
2) Benodigd lokaal team
3) Procedure om implementatie te bewerkstelligen
4) Factsheet
5) Data die nodig zijn om het effect van de implementatie te evalueren

Eerst willen we graag inzicht in de huidige werkwijze.

1. Bent u gewend om SDM toe te passen, waarbij (uitgebreid) aandacht wordt besteed aan PNOM?
   - ☐ Ja, altijd
   - ☐ Ja, soms
   - ☐ Nee

2. Indien nee: waarom niet: ________________________________
   ____

3. Indien ja: hoe vaak in het afgelopen jaar is PNOM middels SDM besproken?
   - ☐ 1 of 2 keer
   - ☐ 3 tot 5 keer
   - ☐ 5 tot 10 keer
   - ☐ 10 keer of vaker

4. Hoe vaak is daarbij voor PNOM gekozen?
   - ☐ 1 of 2 keer
   - ☐ 3 tot 5 keer
   - ☐ 5 tot 10 keer
   - ☐ 10 keer of vaker

5. Indien ja, wat wordt er bij SDM besproken over NOM? ________________________________
   ____

6. Indien ja, wat was uw ervaring met PNOM? ________________________________
   ____

For peer review only - http://bmjopen.bmj.com/site/about/guidelines.xhtml

Datum (dd-mm-jjjj): ___ - ___ - 20___

Wat vindt u van de huidige versie van de PNOM-handleiding? Hebt u aanvullingen of suggesties na lezen ervan? Zo ja, zou u die hieronder willen vermelden?

A) Input voor PNOM-handleiding:

B) Input voor patiëntinformatie:

C) Input voor zakkaart:

D) Input voor factsheet:

E) Overig input:

For peer review only - http://bmjopen.bmj.com/site/about/guidelines.xhtml

Erasmus MC Universitair Medisch Centrum Rotterdam — Amsterdam UMC Universitair Medische Centra — St Antonius een santeon ziekenhuis

Datum (dd-mm-jjjj): ___ - ___ - 20___

Tot slot willen we graag iets meer weten over uzelf.

1. Leeftijd
   - ☐ <30 jaar
   - ☐ 30 t/m 39 jaar
   - ☐ 40 t/m 49 jaar
   - ☐ 50 t/m 59 jaar
   - ☐ 60 jaar of ouder
   - ☐ Wil niet zeggen
2. Geslacht
   - ☐ Man
   - ☐ Vrouw
   - ☐ Wil niet zeggen
3. Functie
   - ☐ Traumachirurg
   - ☐ Orthopedisch chirurg
   - ☐ Geriater
   - ☐ Verpleegkundig specialist
   - ☐ Physician assistant
   - ☐ Manager, namelijk: ____________________
   - ☐ Anders, namelijk: ____________________
4. Religie / geloofsovertuiging ________________________
5. Heeft uw geloofsovertuiging effect op toepassen van PNOM
   - ☐ Ja
   - ☐ Nee
   - ☐ Wil niet zeggen
6. Actieve betrokkenheid bij PNOM na heupfractuur
   - ☐ Ja
   - ☐ Nee

For peer review only

For peer review only - http://bmjopen.bmj.com/site/about/guidelines.xhtml

Erasmus MC Universitair Medisch Centrum Rotterdam
Amsterdam UMC Universitair Medische Centra
St Antonius een santeon ziekenhuis

Datum (dd-mm-jjjj): ___ - ___ - 20___

Datasheet interviews phase 3 (follow-up)

Introductie:
- Deelnemer(s) welkom heten en bedanken voor hun deelname.
- Doel van het interview toelichten.
- Benoemen dat deelname betekent dat de antwoorden gebruikt zullen worden om het PNOM protocol waar mogelijk te verbeteren. Dit zal te allen tijden anoniem zijn. Wie dit niet wil, de kans geven om het gesprek te beëindigen of de bijeenkomst te verlaten.

Uw ziekenhuis neemt deel aan implementatie van PNOM voor een specifieke groep kwetsbare ouderen met een heupfractuur.

Eerst willen we graag weten wat u van de PNOM-implementatie vindt.

1. Hebt u het idee dat door de PNOM implementatie de zorg voor de doelgroep is verbeterd?
   ☐ Ja, de zorg is verbeterd
   ☐ Neutraal
   ☐ Nee, de zorg is verslechterd

2. Indien ja, wat is er verbeterd? ______________________________
   ____

3. Indien nee, wat is er verslechterd? ______________________________
   ____

For peer review only

For peer review only - http://bmjopen.bmj.com/site/about/guidelines.xhtml

Datum (dd-mm-jjjj): ___ - ___ - 20___

Wat vindt u van de huidige versie van de PNOM-handleiding en was is uw ervaring tot nu toe? Als u aanvullingen of suggesties hebt, zou u die hieronder willen vermelden?

A) Dingen die goed gingen:

B) Dingen die minder/niet goed gingen en/of beter moeten:

C) Input voor PNOM-handleiding:

D) Input voor patiëntinformatie:

For peer review only

For peer review only - http://bmjopen.bmj.com/site/about/guidelines.xhtml

Datum (dd-mm-jjjj): ___ - ___ - 20___

E) Input voor zakkaart:

F) Input voor factsheet:

G) Overig input:

H) Benodigdheden om implementatie nog verder te verbeteren:

For peer review only - http://bmjopen.bmj.com/site/about/guidelines.xhtml

Erasmus MC Universitair Medisch Centrum Rotterdam — Amsterdam UMC Universitair Medische Centra — St Antonius een santeon ziekenhuis

ID: PNOM-Implementatie - ____ - ____ Datum (dd-mm-jjjj): ___ - ___ - 20___

Introductie:
- Deelnemer(s) welkom heten en bedanken voor hun deelname.
- Doel van het interview toelichten.
- Benoemen dat deelname betekent dat de antwoorden gebruikt zullen worden in het onderzoek. Dit zal te allen tijden anoniem zijn. Als de gesprekspartner dat niet wil, wordt het gesprek beëindigd.

Tevredenheid van patiënt met de gekozen behandeling

Nadat u uw heup gebroken heeft, is in nauw overleg met de chirurg en klinisch geriater besloten of u geopereerd zou worden of niet.
Om inzicht te krijgen of patiënten ook achteraf tevreden zijn met die oorspronkelijke keuze, willen we u vragen om onderstaande vragen in te vullen.

Indien u **wel** geopereerd bent: Hoe tevreden bent u, op een schaal van 0 to 10, dat u in overleg met de behandelaars besloten heeft om geopereerd te worden?

Indien u **niet** geopereerd bent: Hoe tevreden bent u, op een schaal van 0 to 10, dat u in overleg met de behandelaars besloten heeft om niet geopereerd te worden?

(Graag één antwoord aankruisen; 0 = uiterst ontevreden en 10 = uiterst tevreden)

		**Uiterst Ontevreden**									**Uiterst tevreden**	
		0	1	2	3	4	5	6	7	8	9	10	N.v.t.
1.	Tevredenheid	☐	☐	☐	☐	☐	☐	☐	☐	☐	☐	☐	☐*

* N.v.t. aankruisen indien de naaste deze vraag niet zelf beantwoordt.

De behandelend artsen hebben u (en uw naaste) informatie gegeven over de voordelen en nadelen van een behandeling **met** operatie en van een behandeling **zonder** operatie. Wij willen graag weten wat u van die informatie vond.

2. Wat vond u van de <u>hoeveelheid</u> informatie?
   - ☐ Veel teveel
   - ☐ Een beetje teveel
   - ☐ Precies genoeg
   - ☐ Een beetje te weinig
   - ☐ Veel te weinig

3. Was u tevreden met de <u>inhoud</u> van de informatie?
   - ☐ Ja, alles wat ik wilde weten is besproken
   - ☐ Nee, ik had graag meer willen weten over: ______________________________

4. Vindt u dat bij de keuze van de behandeling voldoende is geluisterd naar wat voor u belangrijk was?
   - ☐ Ja, de artsen hebben samen met mij de beste behandeling gekozen
   - ☐ Nee, er was niet voldoende aandacht voor wat voor mij belangrijk was.

   Als u "nee" hebt aangekruist, kunt u dat toelichten? ______________________________

For peer review only - http://bmjopen.bmj.com/site/about/guidelines.xhtml

Erasmus MC Universitair Medisch Centrum Rotterdam — Amsterdam UMC Universitair Medische Centra — St Antonius een santeon ziekenhuis

ID: PNOM-Implementatie - ____ - ____ **Datum (dd-mm-jjjj): ___ - ___ - 20___**

Introductie:

- Deelnemer(s) welkom heten en bedanken voor hun deelname.
- Doel van het interview toelichten.
- Benoemen dat deelname betekent dat de antwoorden gebruikt zullen worden in het onderzoek. Dit zal te allen tijden anoniem zijn. Als de gesprekspartner dat niet wil, wordt het gesprek beëindigd.

Tevredenheid van naaste met de gekozen behandeling

Nadat uw naaste zijn/haar heup gebroken heeft, is in nauw overleg met de chirurg en klinisch geriater besloten of uw naaste geopereerd zou worden of niet.
Om inzicht te krijgen of patiënten en/of hun naasten ook achteraf tevreden zijn met die oorspronkelijke keuze, willen we u vragen om onderstaande vragen in te vullen.

Indien uw naaste **wel** geopereerd is: Hoe tevreden bent u, op een schaal van 0 to 10, dat in overleg met de behandelaars besloten was dat uw naaste geopereerd zou worden?

Indien uw naaste **niet** geopereerd is: Hoe tevreden bent u, op een schaal van 0 to 10, dat in overleg met de behandelaars besloten was dat uw naaste niet geopereerd zou worden?

(Graag één antwoord aankruisen; 0 = uiterst ontevreden en 10 = uiterst tevreden)

	**Uiterst Ontevreden** 0	1	2	3	4	5	6	7	8	9	**Uiterst tevreden** 10	N.v.t.
1. Tevredenheid	☐	☐	☐	☐	☐	☐	☐	☐	☐	☐	☐	☐*

* N.v.t. aankruisen indien de patiënt deze vraag zelf kan beantwoorden.

De behandelend artsen hebben u informatie gegeven over de voordelen en nadelen van een behandeling **met** operatie en van een behandeling **zonder** operatie. Wij willen graag weten wat u van die informatie vond.

2. Wat vond u van de hoeveelheid informatie?
   - ☐ Veel teveel
   - ☐ Een beetje teveel
   - ☐ Precies genoeg
   - ☐ Een beetje te weinig
   - ☐ Veel te weinig

3. Was u tevreden met de inhoud van de informatie?
   - ☐ Ja, alles wat ik wilde weten is besproken
   - ☐ Nee, ik had graag meer willen weten over:

     ____

4. Vindt u dat bij de keuze van de behandeling voldoende is geluisterd naar wat voor uw naaste belangrijk was?
   Als u "nee" hebt aangekruist, kunt u dat toelichten?
   - ☐ Ja, de artsen hebben samen met mij de beste behandeling voor mijn naaste gekozen
   - ☐ Nee, er was niet voldoende aandacht voor wat voor mijn naaste belangrijk was.

     ____

For peer review only - http://bmjopen.bmj.com/site/about/guidelines.xhtml

ID: PNOM-Implementatie - ____ - ____ **Datum (dd-mm-jjjj): ___ - ___ - 20___**

Aanmeldformulier en contactgegevens nieuwe patiënt

A. Gegevens patiënt

1. Naam: ______________________
2. Adres: ______________________
3. Postcode + woonplaats: ______________________
4. Tel. nummer: ______________________
5. Mobiel nummer: ______________________
6. E-mail: ______________________
7. Includerend ziekenhuis: ______________________
8. PID: ______________________

B. Gegevens alternatieve contactpersoon/wettelijk vertegenwoordiger

1. Naam: ______________________
2. Relatie t.o.v. patiënt: ______________________
3. Adres: ______________________
4. Postcode + woonplaats: ______________________
5. Tel. nummer: ______________________
6. Mobiel nummer: ______________________
7. E-mail: ______________________

C. Akkoord om gegevens met onderzoekers te delen voor belafspraak over 4-6 weken

1. Wie ondertekent voor akkoord? ☐ Patiënt ☐ Wettelijk vertegenwoordiger
2. Datum _____ - _____ - _____ (dd-mm-yyyy)
3. Handtekening ______________________

For peer review only - http://bmjopen.bmj.com/site/about/guidelines.xhtml

Reporting checklist for protocol of a clinical trial.

Based on the SPIRIT guidelines.

Instructions to authors

Complete this checklist by entering the page numbers from your manuscript where readers will find each of the items listed below.

Your article may not currently address all the items on the checklist. Please modify your text to include the missing information. If you are certain that an item does not apply, please write "n/a" and provide a short explanation.

Upload your completed checklist as an extra file when you submit to a journal.

In your methods section, say that you used the SPIRITreporting guidelines, and cite them as:

Chan A-W, Tetzlaff JM, Gøtzsche PC, Altman DG, Mann H, Berlin J, Dickersin K, Hróbjartsson A, Schulz KF, Parulekar WR, Krleža-Jerić K, Laupacis A, Moher D. SPIRIT 2013 Explanation and Elaboration: Guidance for protocols of clinical trials. BMJ. 2013;346:e7586

		Reporting Item	Page Number
**Administrative information**			
Title	#1	Descriptive title identifying the study design, population, interventions, and, if applicable, trial acronym	1

For peer review only

Trial registration	#2a	Trial identifier and registry name. If not yet registered, name of intended registry	3 and 14
Trial registration: data set	#2b	All items from the World Health Organization Trial Registration Data Set	3 and 14
Protocol version	#3	Date and version identifier	3
Funding	#4	Sources and types of financial, material, and other support	14
Roles and responsibilities: contributorship	#5a	Names, affiliations, and roles of protocol contributors	2
Roles and responsibilities: sponsor contact information	#5b	Name and contact information for the trial sponsor	14
Roles and responsibilities: sponsor and funder	#5c	Role of study sponsor and funders, if any, in study design; collection, management, analysis, and interpretation of data; writing of the report; and the decision to submit the report for publication, including whether they will have ultimate authority over any of these activities	14
Roles and responsibilities: committees	#5d	Composition, roles, and responsibilities of the coordinating centre, steering committee, endpoint adjudication committee, data management team, and	7-14

For peer review only

		other individuals or groups overseeing the trial, if applicable (see Item 21a for data monitoring committee)	
**Introduction**			
Background and rationale	#6a	Description of research question and justification for undertaking the trial, including summary of relevant studies (published and unpublished) examining benefits and harms for each intervention	5-6
Background and rationale: choice of comparators	#6b	Explanation for choice of comparators	5-6
Objectives	#7	Specific objectives or hypotheses	6
Trial design	#8	Description of trial design including type of trial (eg, parallel group, crossover, factorial, single group), allocation ratio, and framework (eg, superiority, equivalence, non-inferiority, exploratory)	5-6
**Methods: Participants, interventions, and outcomes**			
Study setting	#9	Description of study settings (eg, community clinic, academic hospital) and list of countries where data will be collected. Reference to where list of study sites can be obtained	7

For peer review only

Eligibility criteria	#10	Inclusion and exclusion criteria for participants. If applicable, eligibility criteria for study centres and individuals who will perform the interventions (eg, surgeons, psychotherapists)	8
Interventions: description	#11a	Interventions for each group with sufficient detail to allow replication, including how and when they will be administered	8-13
Interventions: modifications	#11b	Criteria for discontinuing or modifying allocated interventions for a given trial participant (eg, drug dose change in response to harms, participant request, or improving / worsening disease)	8-12
Interventions: adherance	#11c	Strategies to improve adherence to intervention protocols, and any procedures for monitoring adherence (eg, drug tablet return; laboratory tests)	8-14
Interventions: concomitant care	#11d	Relevant concomitant care and interventions that are permitted or prohibited during the trial	NA
Outcomes	#12	Primary, secondary, and other outcomes, including the specific measurement variable (eg, systolic blood pressure), analysis metric (eg, change from baseline, final value, time to event), method of aggregation (eg, median, proportion), and time point for each outcome. Explanation of the clinical relevance of chosen efficacy and harm outcomes is strongly recommended	11-12

For peer review only

Participant timeline	#13	Time schedule of enrolment, interventions (including any run-ins and washouts), assessments, and visits for participants. A schematic diagram is highly recommended (see Figure)	8-11
Sample size	#14	Estimated number of participants needed to achieve study objectives and how it was determined, including clinical and statistical assumptions supporting any sample size calculations	12
Recruitment	#15	Strategies for achieving adequate participant enrolment to reach target sample size	9-12
**Methods: Assignment of interventions (for controlled trials)**			
Allocation: sequence generation	#16a	Method of generating the allocation sequence (eg, computer-generated random numbers), and list of any factors for stratification. To reduce predictability of a random sequence, details of any planned restriction (eg, blocking) should be provided in a separate document that is unavailable to those who enrol participants or assign interventions	NA
Allocation concealment mechanism	#16b	Mechanism of implementing the allocation sequence (eg, central telephone; sequentially numbered, opaque,	NA

For peer review only

For peer review only

		sealed envelopes), describing any steps to conceal the sequence until interventions are assigned	
Allocation: implementation	#16c	Who will generate the allocation sequence, who will enrol participants, and who will assign participants to interventions	NA
Blinding (masking)	#17a	Who will be blinded after assignment to interventions (eg, trial participants, care providers, outcome assessors, data analysts), and how	NA
Blinding (masking): emergency unblinding	#17b	If blinded, circumstances under which unblinding is permissible, and procedure for revealing a participant's allocated intervention during the trial	NA
**Methods: Data collection, management, and analysis**			
Data collection plan	#18a	Plans for assessment and collection of outcome, baseline, and other trial data, including any related processes to promote data quality (eg, duplicate measurements, training of assessors) and a description of study instruments (eg, questionnaires, laboratory tests) along with their reliability and validity, if known. Reference to where data collection forms can be found, if not in the protocol	11-13

Data collection plan: retention	#18b	Plans to promote participant retention and complete follow-up, including list of any outcome data to be collected for participants who discontinue or deviate from intervention protocols	NA
Data management	#19	Plans for data entry, coding, security, and storage, including any related processes to promote data quality (eg, double data entry; range checks for data values). Reference to where details of data management procedures can be found, if not in the protocol	13
Statistics: outcomes	#20a	Statistical methods for analysing primary and secondary outcomes. Reference to where other details of the statistical analysis plan can be found, if not in the protocol	12-13
Statistics: additional analyses	#20b	Methods for any additional analyses (eg, subgroup and adjusted analyses)	13
Statistics: analysis population and missing data	#20c	Definition of analysis population relating to protocol non-adherence (eg, as randomised analysis), and any statistical methods to handle missing data (eg, multiple imputation)	NA

Methods: Monitoring

Data monitoring: formal committee	#21a	Composition of data monitoring committee (DMC); summary of its role and reporting structure; statement of whether it is independent from the sponsor and competing interests; and reference to where further	13

For peer review only

		details about its charter can be found, if not in the protocol. Alternatively, an explanation of why a DMC is not needed	
Data monitoring: interim analysis	#21b	Description of any interim analyses and stopping guidelines, including who will have access to these interim results and make the final decision to terminate the trial	NA
Harms	#22	Plans for collecting, assessing, reporting, and managing solicited and spontaneously reported adverse events and other unintended effects of trial interventions or trial conduct	13-14
Auditing	#23	Frequency and procedures for auditing trial conduct, if any, and whether the process will be independent from investigators and the sponsor	NA
**Ethics and dissemination**			
Research ethics approval	#24	Plans for seeking research ethics committee / institutional review board (REC / IRB) approval	13-14
Protocol amendments	#25	Plans for communicating important protocol modifications (eg, changes to eligibility criteria, outcomes, analyses) to relevant parties (eg, investigators, REC / IRBs, trial participants, trial registries, journals, regulators)	NA

For peer review only

Consent or assent	#26a	Who will obtain informed consent or assent from potential trial participants or authorised surrogates, and how (see Item 32)	13-14
Consent or assent: ancillary studies	#26b	Additional consent provisions for collection and use of participant data and biological specimens in ancillary studies, if applicable	NA
Confidentiality	#27	How personal information about potential and enrolled participants will be collected, shared, and maintained in order to protect confidentiality before, during, and after the trial	13-14
Declaration of interests	#28	Financial and other competing interests for principal investigators for the overall trial and each study site	14
Data access	#29	Statement of who will have access to the final trial dataset, and disclosure of contractual agreements that limit such access for investigators	13
Ancillary and post trial care	#30	Provisions, if any, for ancillary and post-trial care, and for compensation to those who suffer harm from trial participation	NA
Dissemination policy: trial results	#31a	Plans for investigators and sponsor to communicate trial results to participants, healthcare professionals, the public, and other relevant groups (eg, via publication, reporting in results databases, or other data sharing arrangements), including any publication restrictions	13-14

For peer review only

Dissemination policy: authorship	#31b	Authorship eligibility guidelines and any intended use of professional writers	NA
Dissemination policy: reproducible research	#31c	Plans, if any, for granting public access to the full protocol, participant-level dataset, and statistical code	14

Appendices

Informed consent materials	#32	Model consent form and other related documentation given to participants and authorised surrogates	NA
Biological specimens	#33	Plans for collection, laboratory evaluation, and storage of biological specimens for genetic or molecular analysis in the current trial and for future use in ancillary studies, if applicable	NA

The SPIRIT Explanation and Elaboration paper is distributed under the terms of the Creative Commons Attribution License CC-BY-NC. This checklist was completed on 18. December 2023 using https://www.goodreports.org/, a tool made by the EQUATOR Network in collaboration with Penelope.ai

For peer review only
